# Expanding Public Access to Understanding Data: A Case Study of Leveraging Generative AI for India Policy Insights Dashboard

**DOI:** 10.1080/00330124.2026.2667385

**Published:** 2026-06-01

**Authors:** Sandesh Sharma Dulal, Devika Jain, Zachary Sherman, Mengxi Zhang, Rockli Kim, S V Subramanian, Junghwan Kim

**Affiliations:** a Department of Geography, Virginia Tech, Blacksburg, VA, USA; b Center for Geographic Analysis, Harvard University, Cambridge, MA, USA; c Department of Health Systems and Implementation Science, Virginia Tech Carilion School of Medicine, Roanoke, VA, USA; d Division of Health Policy and Management, Korea University, Seoul, South Korea; e Harvard Center for Population and Development Studies, Cambridge, MA, USA

**Keywords:** chatbot, dashboard, Generative AI, India Policy Insights (IPI), Large Language Model (LLM)

## Abstract

Interactive dashboards have become central tools for visualizing policy-relevant data, but they remain limited by rigid structures and technical complexity. Recent advances in large language model (LLM)-powered conversational AI offer a way to address these challenges, as LLMs can interpret unstructured natural language inputs and translate them into structured functions. This proof-of-concept study introduces a Generative AI (GenAI)-powered chatbot that integrates LLMs with an online dashboard, demonstrated through the India Policy Insights (IPI) dashboard. The system translates conversational queries into structured functions, retrieves validated outputs from a spatially enabled database, and presents results as text, charts, and maps. We implemented 13 representative functions spanning spatial, temporal, composite, classification, and constraint-based analyses. Results show that spatial, classification, and constraint-based functions achieved consistently high accuracy due to explicit parameters, while composite multi-indicator functions posed greater challenges. The findings demonstrate the potential of GenAI-powered interactive dashboards to support language-driven interaction and broaden accessibility for diverse user groups, regardless of technological literacy. Beyond the case study, the proposed framework provides a design pathway for developing GenAI-powered dashboards that democratize access to spatiotemporal data, enhance evidence-based policymaking, and help bridge persistent gaps between data and actionable insights.

## 1. Introduction

In recent years, data visualization dashboards have emerged as a central tool for communicating information on key policy indicators. Governments, researchers, and civil society organizations have widely adopted dashboards to monitor development trends, track health outcomes, and inform decision-making across domains ranging from education, sanitation, to poverty, and climate governance [1]. The COVID-19 pandemic significantly accelerated this trajectory when many governments developed real-time dashboards to track cases, mortality, vaccination rates, and mobility patterns [2]. Civil society and media outlets complemented these efforts with independent trackers, which rapidly became primary sources of public information [3]. This proliferation underscored the centrality of dashboards as the dominant mode of presenting complex datasets to diverse audiences.

The widespread use of dashboards is attributable to several advantages. They provide centralized access to heterogeneous datasets, transform complex indicators into interpretable visualizations, and promote transparency through open, interactive interfaces [4], [5], [6]. Well-designed dashboards enhance comparability across geographies and over time, while reinforcing accountability by making information publicly accessible. Their visual and interactive affordances have rendered them indispensable in the policymaker’s toolkit, as well as a critical mechanism for public engagement.

However, the effectiveness of dashboards is often constrained by their fundamental design limitation: they assume users are already familiar with the structure of indicators, the geographic boundaries of administrative units, and the technical language of data analytics [7], [8]. Each visualization is inherently editorialized, reflecting the choices of the developer regarding which indicators to include, how to structure them, and what trends to foreground [9]. As a result, users are restricted to the questions and outputs anticipated by the dashboard’s architecture, limiting the scope for nuanced or context-specific inquiries [10]. Moreover, effective navigation often presupposes a degree of digital literacy for selecting appropriate indicators, applying filters, and interpreting spatial or statistical representations [11], [12]. Even well-crafted dashboards can become cumbersome to use, as their “one-size-fits-all” structure necessarily simplifies the diversity of user needs [13]. Thus, while dashboards appear to democratize access to data, in practice, they cannot fully accommodate the wide spectrum of users.

Recent advances in artificial intelligence, such as Large Language Models (LLMs), provide an opportunity to overcome these constraints. Unlike traditional dashboard interfaces that rely on rigid filters, dropdown menus, and predefined visualizations, LLMs enable natural language interaction with data systems [14], [15], [16], [17]. Users can pose questions in plain language and receive coherent, contextually relevant responses [17]. These responses can be delivered in multiple modalities, including narrative text, statistical outputs, and geospatial visualizations [18], [19], [20], [21]. This model of interaction removes the prerequisite of digital literacy. Just as individuals learned to navigate search engines and social media platforms without formal instruction, GenAI can lower barriers to engagement and allows data systems to be genuinely user-driven [22], [23], [24], [25], [26], [27]. These studies demonstrate the potential of LLMs to lower technical barriers by translating natural-language inputs into executable spatial operations. However, many of these works treat conversational GIS as a general-purpose spatial query problem, often focusing on open-ended datasets or developer-oriented tool use.

In contrast to current conversational GIS tools, our study advances a new paradigm for equitable engagement with policy-relevant data powered by GenAI. By developing a platform that integrates GenAI with interactive map dashboards, we seek to expand the capacity of diverse populations to meaningfully engage with critical public issues such as health, sanitation, nutrition, and education, which directly affect their well-being [28], [29]. This study contributes to the emerging literature on LLM-GIS integration by focusing on the practical deployment of conversational AI within a real-world, policy-oriented dashboard environment. Rather than advancing a new conversational GIS framework, the contribution lies in demonstrating how existing AI and GIS components can be combined into a coherent, deployable workflow that addresses concrete usability and accessibility challenges associated with traditional dashboards. We describe its design and functionality, assess its potential for inclusive policy engagement, and present it as proof-of-concept for applying conversational AI in public health.

**Figure 1.**
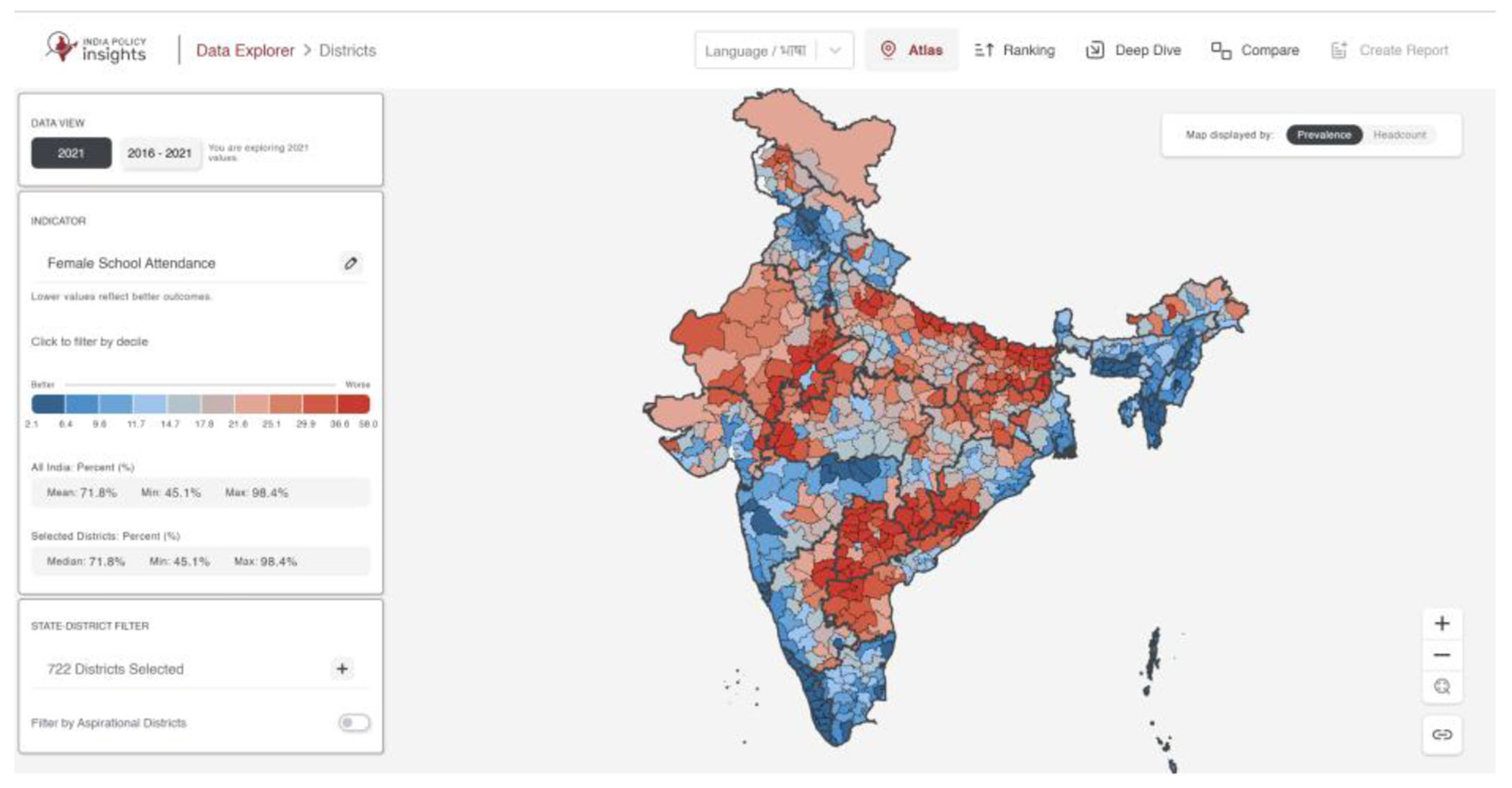
Screenshot of the India Policy Insights interactive online dashboard [31].

Specifically, this case study aims to enhance the India Policy Insights (IPI) dashboard (Figure 1) with a chatbot to democratize access to data for policymakers, NGOs, and the public through GenAI. The IPI platform, developed by the Geographic Insights Lab (GIL) at Harvard University, is a spatiotemporal visualization system that provides policymakers with granular, data-driven insights into health disparities across India. It visualizes 122 health and social determinants of health indicators across 720 districts and 543 parliamentary constituencies [30]. Although the IPI dashboard has strong potential to assess and address local challenges with precision [31], its complexity makes it difficult to navigate for stakeholders with limited technological literacy, much like other conventional online dashboards.

The IPI case provides an opportunity to illustrate the added value of conversational AI when embedded within an interactive geospatial dashboard and to evaluate its performance across different query types, levels of specificity, and degrees of complexity. Moreover, applications that integrate AI-driven analytics with dashboard-based geospatial visualization in non-Western policy contexts remain relatively limited in the literature; this case therefore broadens the geographic and institutional scope of existing research.

The system’s core functions include query interpretation, database interaction, and output visualization. While the current prototype demonstrates foundational capabilities, future production-ready versions must support broader queries, richer visualizations, scalability, and enhanced user experience features such as personalization, multilingual support, and mobile responsiveness. Additionally, transitioning to cost-effective open-source models and implementing robust backend measures for security, access control, and data privacy will be essential for real-world deployment and sustainable scalability.

Beyond this practical contribution, we also aim to advance the emerging literature on the integration of GIS and GenAI by demonstrating how these two elements can be effectively combined, considering multiple factors that may influence the performance of chatbot-based dashboards and, ultimately, affect user acceptance and application. To this end, we conducted systematic evaluations of the developed chatbot’s performance across multiple dimensions to contribute to a broader understanding of design considerations for GenAI-enabled dashboards. The results of the systematic evaluation of the chatbot’s performance provide valuable lessons for developers seeking to build GenAI-powered dashboards similar to ours.

## 2. Methods

### 2.1 Architecture of the chatbot

**Figure 2.**
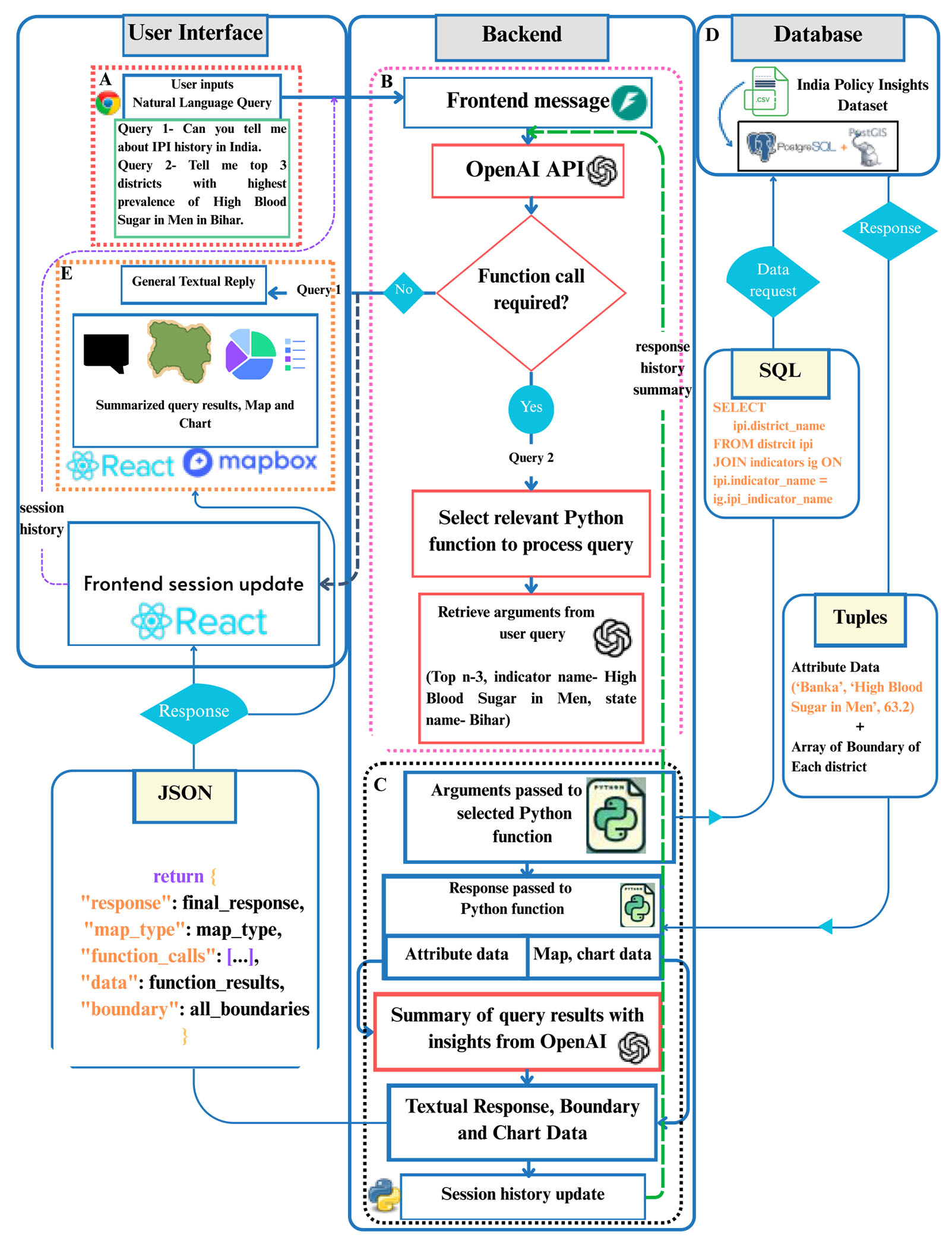
System architecture. (A) User interface. (B) The backend process with OpenAI interaction. (C) Functions executing queries against the database (D), returning attribute and boundary data. (E) The final responses displayed to the user.

Figure 2 presents the full execution workflow of the system, starting with a user’s natural language query and ending with the delivery of results in text, chart, and map formats. In step (A), the user submits a query through the frontend interface, such as “Tell me about Sustainable Development Goals” or “Map the top three districts with the highest prevalence of high blood sugar in men in Bihar.” This request is passed to the backend, where FastAPI (a Python-based library for data validation that uses JSON schema) [32] receives and processes the message.

In step (B), once FastAPI forwards the query to the OpenAI API [33], the API determines whether the query requires a function call [34]. Function calling involves extracting relevant parameters (e.g., indicator name, geography, year, and scope) from natural language and invoking predefined backend functions with these structured inputs. For example, if the query involves structured analytics, spatial data retrieval, or calculations (e.g., “Map the top three districts with the highest prevalence of high blood sugar in men in Bihar”), the LLM extracts “indicator: High Blood Sugar in Men, geography: Bihar, top_n: 3” and passes them to the appropriate Python function. However, if the user’s question can be answered directly without invoking predefined functions (e.g., “Tell me about SDG history in India”), the model provides a text-based response without engaging backend functions.

In step (C), the backend Python function uses the extracted parameters to execute an SQL (Structured Query Language) query against the PostGIS and PostgreSQL databases [35], [36].

Step (D) represents the database layer, where the datasets are stored. Queries to this layer return both numeric results and geographic boundaries, ensuring that the outputs are suitable for visualization. These raw outputs are then transformed into a structured JSON format that includes attribute data, map-ready boundary information, and chart metadata. The attribute data are further processed with an LLM, which generates a natural language description of the results. All outputs are returned to the frontend as a JSON response containing *response* (i.e., generated text), *map_type* (i.e., visualization type), *data* (i.e., attribute values and analysis), and *boundary* (i.e., spatial geometries).

In step (E), the frontend renders these outputs as a coherent and user-friendly response that includes a textual explanation, a map (via Mapbox [37]), and charts (via React components [38]).

### 2.2 Featured queries of the chatbot

We designed 13 functions to address queries ranging from those posed by the general public to those relevant to policymakers, drawing on the local knowledge and context gained from our previous work. These functions can be broadly categorized into five groups: (1) spatial, (2) temporal and comparative change analysis, (3) composite or multi-indicator analysis, (4) classification and similarity analysis, and (5) constraint-based analysis. A detailed explanation of these categories is provided in the Supplementary Section.

### 2.3 Evaluation of the chatbot’s performance

The evaluation of our chatbot’s performance in terms of accuracy begins with measuring how reliably OpenAI’s LLM maps a user prompt to the correct function signature. The overall accuracy of the system depends on the function-calling accuracy of the LLM. Once the correct function is called, the resulting outputs (e.g., attributes, maps, charts) are accurate because they are generated by predefined programs that were developed and validated by the research team. In other words, if the model triggers the wrong function or passes incorrect arguments, the downstream SQL query, map rendering, and chart generation will also be incorrect. Therefore, our evaluation focused on whether the chatbot correctly triggered predefined functions.

**Figure 3.**
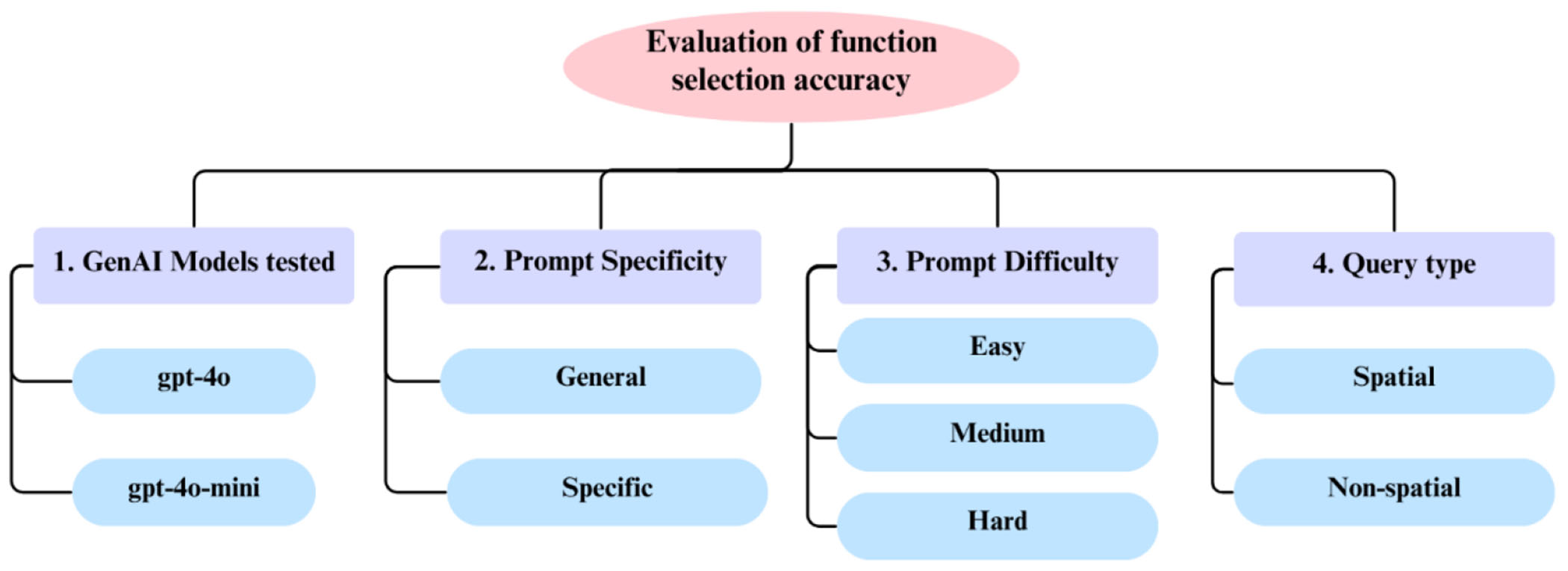
Overview of the chatbot evaluation approach.

To assess function selection accuracy, we adopted and adjusted a two-axis evaluation approach used in previous studies [39], [40]. Specifically, we evaluated 12 different functions across four dimensions: (1) GenAI model (GPT-4o vs. GPT-4o-mini), (2) prompt specificity (general vs. specific), (3) prompt difficulty (easy, medium, hard), and (4) query type (spatial vs. non-spatial). Although there are 13 functions implemented in the application, we evaluated 12 functions as two functions -- District Classification (classifying districts into a number of classes based on prevalence value) and District Classification Change (classifying districts into a number of classes based on prevalence change value) -- have the same characteristics and parameters and hence the queries would be very similar to each other. A comprehensive list of testing queries is provided in the Supplementary Information. Figure 3 illustrates the evaluation approach.

#### 2.3.1 Evaluation of prompt specificity (general vs. specific)

Evaluation prompts were prepared and divided into two groups: general (i.e., conversational and underspecified) and specific (i.e., queries in which nearly all function parameters are explicitly provided). We created 20 testing queries per function, with 10 general and 10 specific prompts, for evaluation.

(1) General prompts simulate loosely structured, conversational user queries where the LLM must infer missing parameters necessary to execute the function. For example, “Who leads on average performance along the Tamil Nadu edge?” is a general prompt. This type of test provides a comprehensive measure of the model’s ability to handle vague queries and generate meaningful results despite missing input components.

(2) Specific prompts contain all parameters explicitly required to execute the function. For instance, “Report anemia among women and under-five stunting in districts bordering Gujarat for 2016” is a specific prompt. Unlike the general query, the specific query specifies the health indicators (“anemia among women” and “under-five stunting”) and the data year (“2016”).

#### 2.3.2 Evaluation of prompt difficulty (easy, medium, hard)

The evaluation followed a three-tiered framework, adapted from ToolACE for realistic tool-use evaluation [41]. Prompt difficulty increases with the number of parameters, as well as the complexity and scope of the analysis. Prompt difficulty is defined with respect to the interaction between the language model and the system’s tool interface, rather than the intrinsic computational complexity of the data retrieval or aggregation procedures. Specifically, difficulty reflects the number of parameters, constraints, and reasoning steps the model must jointly interpret and compose to generate a valid and complete function call. For evaluation, we designed 15 testing queries per function, allocating five to each tier: easy, medium, and hard.

(1) Easy-level prompts are basic queries that use only the required fields, such as a single place or a single indicator. For example, “Provide diabetes prevalence for Surat” represents an easy query.

(2) Medium-level prompts add one additional detail (e.g., year, benchmark, state filter, or simple spatial radius) to the easy-level query. Example prompts include “Compare Mumbai and Delhi on institutional delivery rate using national averages” or “For 2021, report neonatal mortality rate for Varanasi.”

(3) Hard-level prompts combine two or more additional parameters and expand the scope to multiple districts and indicators, often involving spatial filtering, benchmarking, or geometry toggles. Example includes “Starting from Hyderabad, analyze exclusive breastfeeding, ANC four or more visits, and postnatal care within 48 hours for districts within 180 km; return up to 30 districts and omit boundary geometry.”

#### 2.3.3 Evaluation of GenAI models (GPT-4o vs. GPT-4o-mini)

Each testing query was executed with two OpenAI GenAI models: GPT-4o-mini and GPT-4o. GPT-4o and GPT-4o-mini are among the most popular models used for a wide variety of works due to their efficiency and high performance [42], [43]. We chose the OpenAI API because it is developer-friendly, with simple endpoints and clear documentation that enable quick and seamless integration into our system [33]. GPT-4o-mini is categorized as a Small Language Model (SLM), optimized for efficiency with fewer parameters and lower computational overhead. It is designed to deliver fast responses with minimal latency while consuming fewer computing resources [44]. In contrast, GPT-4o is a Large Language Model (LLM) with a substantially higher parameter count, which results in greater accuracy and reasoning ability but also higher resource consumption. While large language models generally outperform small models in accuracy and reasoning tasks, they require significantly more memory and computational resources for both training and inference [44]. Furthermore, although complex models are often assumed to consistently outperform smaller models, performance can vary depending on task-specific contexts. Therefore, it is important to examine how the two GenAI models differ in handling prompts of varying complexity.

#### 2.3.4 Evaluation of query types (spatial vs. non-spatial)

We evaluated the chatbot’s accuracy across three spatial and nine non-spatial query types. Spatial queries capture geographic relationships such as distance, coordinates, and geometric relations (e.g., touching or adjacency) [17], [45]. Other functions are non-spatial and include pure retrieval, ranking, constraints, composites, change analysis, and classification. Because spatial querying is a core capability for the future use of the chatbot, it is important to assess performance across both spatial and non-spatial tasks.

## 3. Results

### 3.1 Representative features of the developed chatbot

This section presents an illustrative example of a key function (“Get districts within a given radius”) to demonstrate how users interact with the chatbot. The online chatbot is available at the following link (https://ipipoc.netlify.app/). Additional features of the chatbot are provided in the Supplementary Information.

#### 3.1.1 Get districts within a given radius

**Figure 4.**
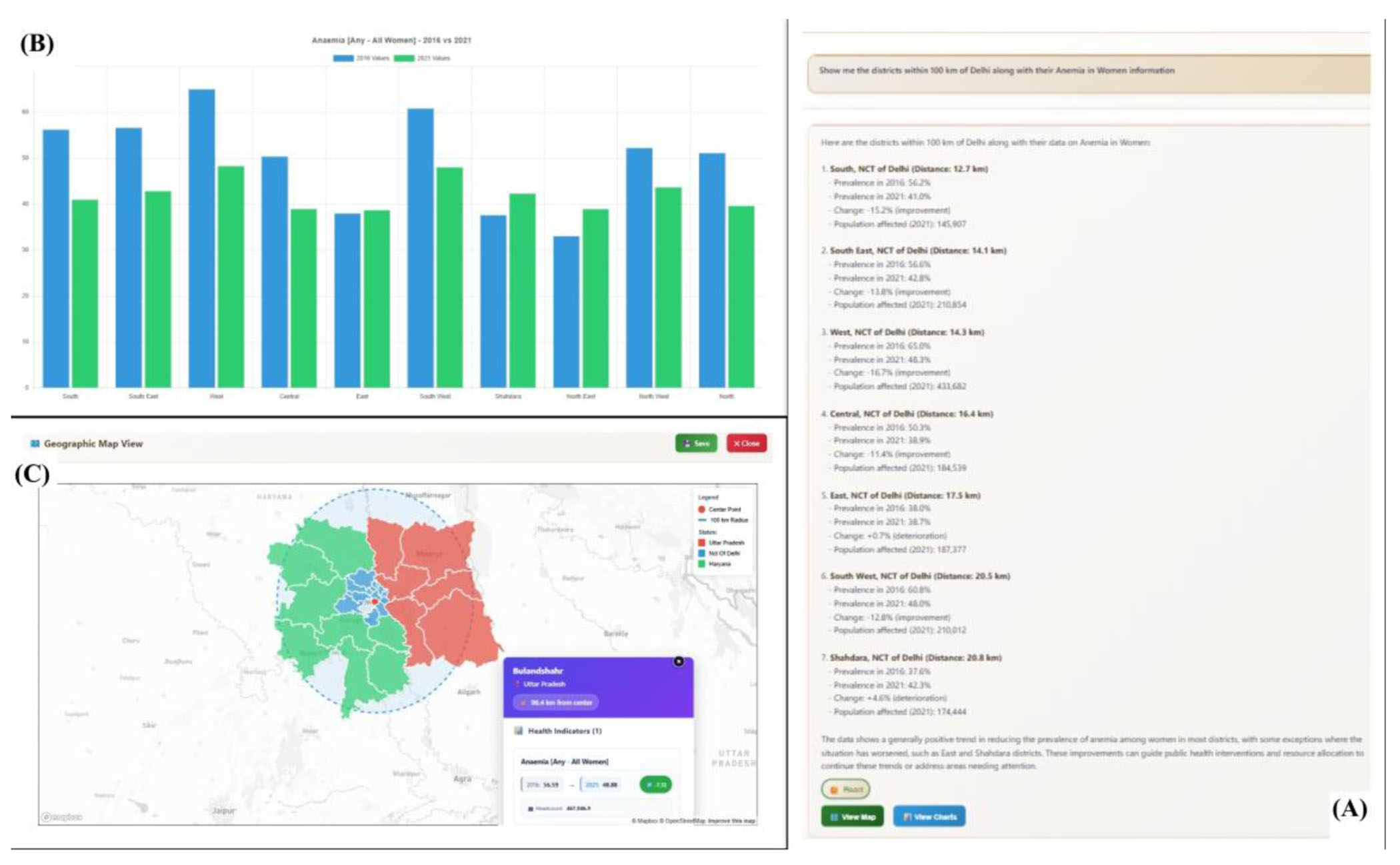
The chatbot’s output for the prompt: “Show me the districts within 100 km of Delhi along with their Anemia in Women information.” (A) Text summary listing districts within 100 km of Delhi with prevalence values, temporal changes, and estimated affected populations. (B) Bar chart comparing prevalence levels across districts and years. (C) Interactive map showing district boundaries and the spatial distribution of prevalence relative to the center point.

As shown in Figure 4(A), the chatbot provides a detailed textual breakdown that lists each district within the specified radius, along with its distance from the center point and anemia prevalence values for 2016 and 2021, including percentage change and estimated affected population. For this query, the LLM extracts the center point, radius, and indicator name from the user prompt and maps them to the corresponding system function parameters (details provided in the Supplementary Material). When no temporal range is specified, the system defaults to returning values for 2021 for the selected indicator. Second, the function generates comparative bar charts (Figure 4(B)) that visualize temporal changes in prevalence across multiple districts. Finally, an interactive map (Figure 4(C)) displays the spatial distribution of districts relative to the center point, using color coding and distance markers to contextualize the statistics geographically.

### 3.2 Results on the evaluation of the chatbot’s performance

#### 3.2.1 Evaluation based on prompt specificity (general vs. specific)

**Figure 5.**
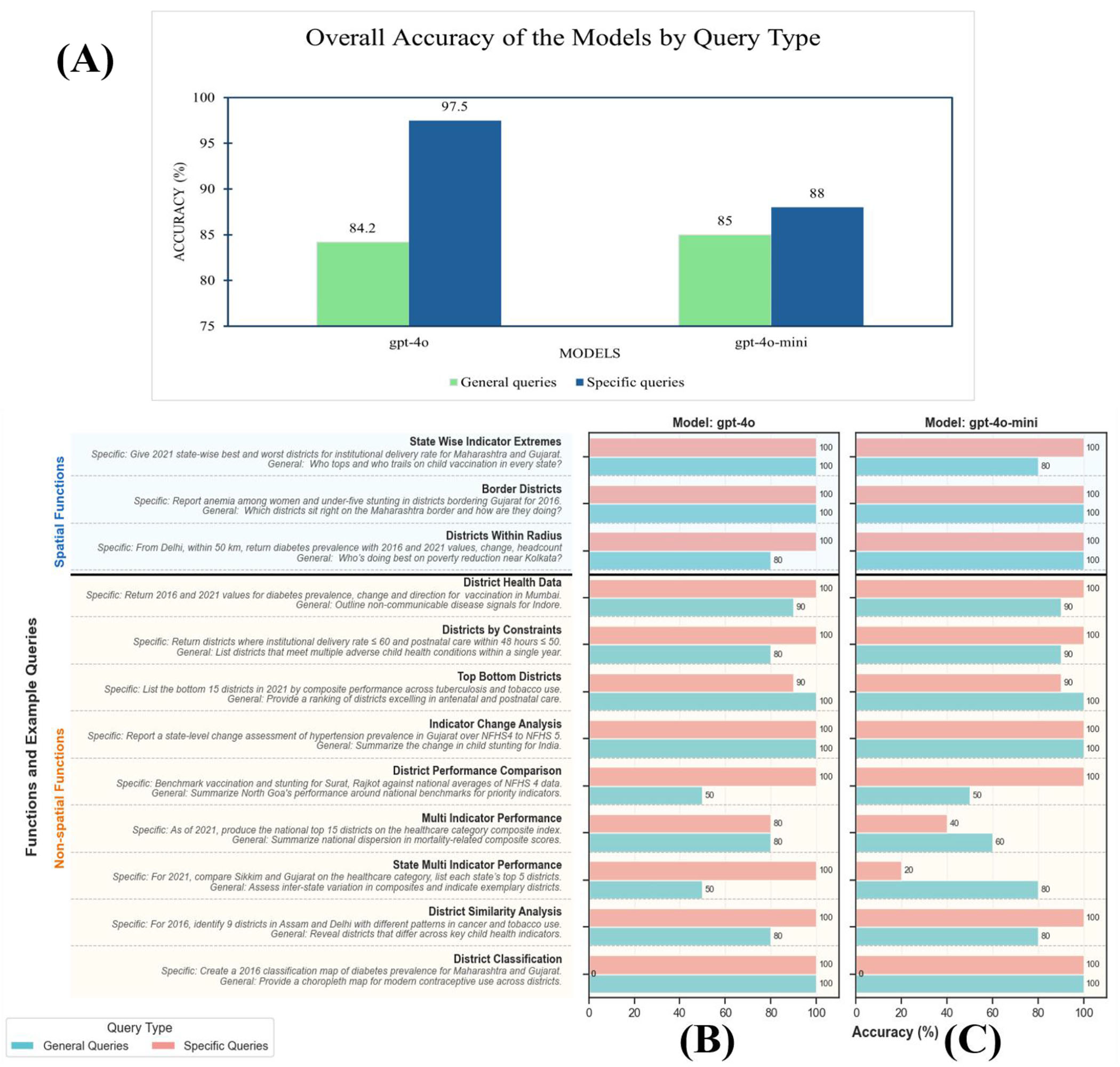
(A) Results on the overall function-call accuracy of the GPT-4o and GPT-4o-mini based on the query specificity (general versus specific queries). Function call accuracy by query type (General/Specific) of (B) GPT-4o and (C) GPT-4o-mini.

Figure 5(A) compares the chatbot’s performance in triggering correct function calls for GPT-4o and GPT-4o-mini when tested with general and specific prompts. In both models, specific prompts yield higher accuracy because they explicitly include parameters such as the goal, year, and indicator(s). When these parameters are clearly stated, the model can more easily select the appropriate function and return the correct result. Overall, GPT-4o shows the greatest improvement, with accuracy increasing from about 84 percent on general prompts to 97 percent on specific ones. GPT-4o-mini shows a smaller gain, improving from roughly 85 percent to 88 percent.

Next, we compare the performance of GPT-4o-mini on spatial and non-spatial queries (Figure 5(B) and 5(C)). For spatial queries, accuracy remains consistently high (80 percent to 100 percent) for both general and specific prompts. In contrast, the accuracy of non-spatial queries is more varied across different tasks. For example, “Indicator Change Analysis,” which compares changes in indicator values between two years, and “District Classification,” which categorizes and maps districts by a single indicator value, both achieve 100 percent accuracy for general and specific prompts. By contrast, tasks that require more complex composition or category resolution perform poorly. Specifically, the “Multi Indicator Performance” query, which builds a normalized multi-indicator composite and ranking, and the “State Multi Indicator Performance” query, which generates state-level composites with top and bottom districts, show substantially lower accuracy (20 percent to 40 percent) for specific queries and (60 percent to 80 percent) for general queries. When comparing GPT-4o with GPT-4o-mini, GPT-4o shows a smaller gap between general and specific queries and demonstrates greater robustness across both spatial and non-spatial tasks, although “multi-indicator” queries remain the most challenging. For example, in the case of the “State Multi Indicator Performance” and “Multi Indicator Performance” functions, GPT-4o-mini achieved only 20 percent and 40 percent accuracy, respectively, even for specific queries. By contrast, GPT-4o achieved 100 percent accuracy for the former and 80 percent for the latter.

#### 3.2.2 Evaluation based on prompt difficulty (easy vs. medium vs. hard)

**Figure 6.**
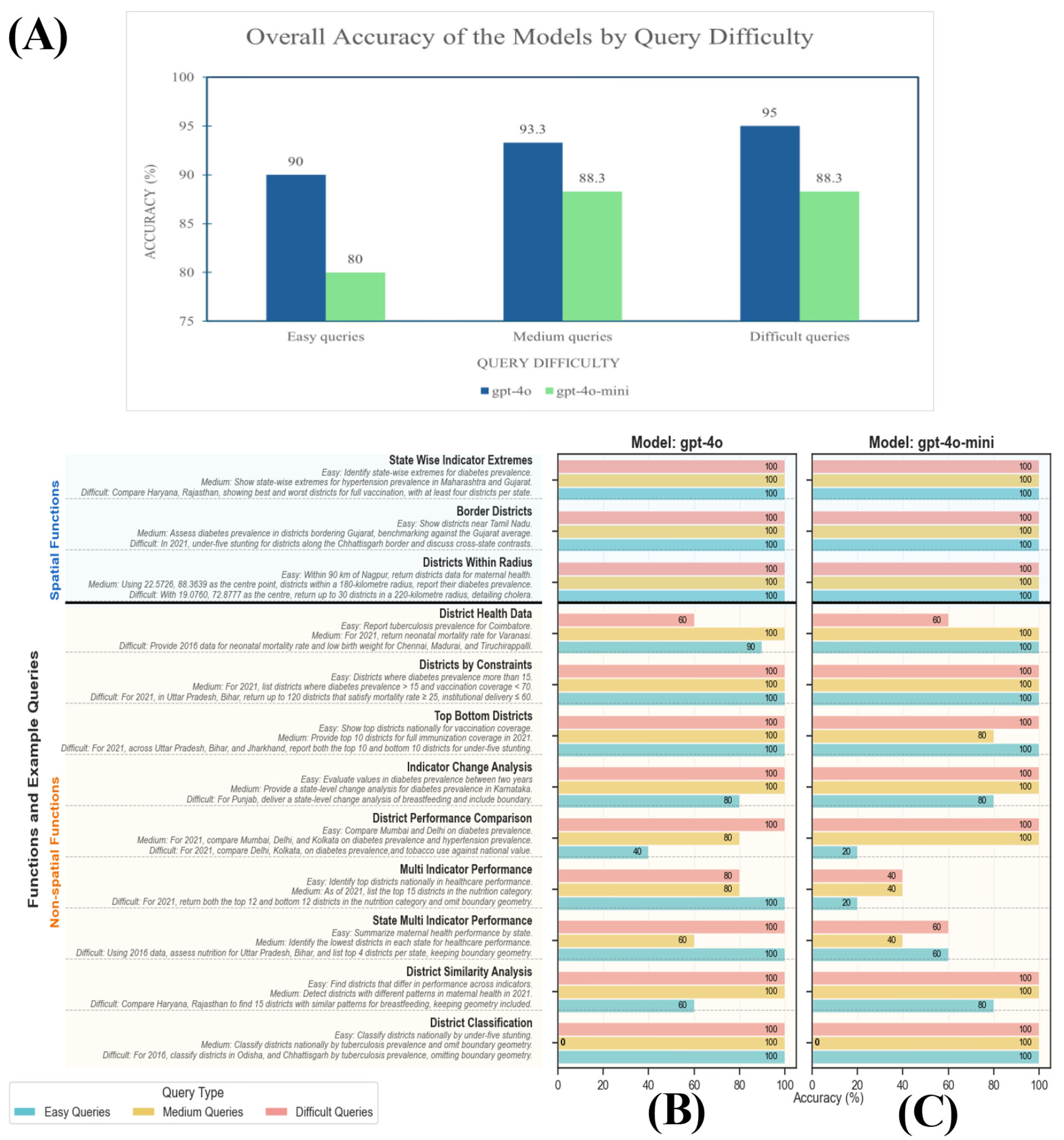
(A) Results on the overall function-call accuracy of the gpt-4o and gpt-4o-mini based on the query specificity (easy vs medium vs hard). Function call accuracy by query type (Easy/Medium/Hard) of (B) gpt-4o and (C) gpt-4o-mini.

Figure 6(A) compares the chatbot’s performance in triggering correct function calls for GPT-4o and GPT-4o-min when tested with three levels of prompt difficulty: easy, medium, and hard. For both models, accuracy increases slightly with difficulty, as parameter-rich prompts provide more structure for mapping onto the correct functions. For example, in the case of easy queries, GPT-4o and GPT-4o-mini achieved 90 percent and 80 percent accuracy, respectively, whereas for hard queries, the accuracy rose to 95 percent for GPT-4o and 88.3 percent for GPT-4o-mini. Overall, across all difficulty levels, GPT-4o consistently outperforms GPT-4o-mini.

We further compared GPT-4o-mini’s performance across spatial and non-spatial queries of varying difficulty (Figure 6(B) and 6(C)). For spatial functions, accuracy remains consistently high regardless of difficulty level. For non-spatial functions, however, accuracy varies more noticeably with query complexity. Functions such as Districts by Constraints (which filter districts by threshold values of indicators) show perfect accuracy across all difficulty levels. By contrast, functions such as Multi-Indicator Performance (which generate a composite index across multiple indicators) and State Multi-Indicator Performance (which compute state-level composites with district context) show low-to-moderate accuracy, ranging between 20 percent and 60 percent.

In the case of GPT-4o, the spatial queries showed perfect accuracy of 100 percent across all difficulty levels, alongside some non-spatial functions like District Classification, Districts by Constraints, and Top Bottom Districts. The weakest results were observed for District Health Data (which returns indicator data for one or more districts) and District Performance Comparison (which benchmarks districts against state or national averages), both of which reported 60 percent accuracy for hard queries and 40 percent accuracy for easy queries, respectively. The loss in accuracy is due to the reciprocal misrouting of the functions.

## 4. Discussion

In this proof-of-concept study, we focus on the India Policy Insights (IPI) datasets, which contain a wide range of public policy indicators (e.g., health, nutrition, and population measures) across multiple spatial scales in India, including districts, parliamentary constituencies, assembly constituencies, and villages. An interactive dashboard for the IPI datasets has already been developed [46]. However, navigating the full set of dashboard features may not be equally accessible to all users, particularly those unfamiliar with complex filters and spatial interfaces [11], [12], [13]. This practical challenge highlights the need for more accessible and inclusive approaches to online dashboards and provides an ideal use-inspired research context for conducting proof-of-concept work. To address this need, we proposed and tested a chatbot powered by GenAI, which translates natural language queries into meaningful outputs, delivers contextual interpretations of results, and generates corresponding maps and charts. Although this case study focuses on the India Policy Insights dashboard, the overall framework is independent and modular. Adapting the system to other regions primarily requires replacing the underlying dataset and indicator catalog, while reusing the same LLM-based query interpretation, function-calling logic, and visualization pipeline. Because geographic resolution, indicator metadata, and backend functions are decoupled from the frontend and orchestration layers, the architecture can be readily extended to other national or subnational policy dashboards with minimal changes to the core system.

Overall, the performance comparison across functions shows that spatial, classification, and constraint-based analyses achieve significantly higher accuracy than other categories. This is because their parameters are highly explicit and map directly to function inputs. For example, spatial queries specify clear geographic elements such as coordinates, radius, or border states. Similarly, classification queries are triggered by explicit requests for categorization. These well-defined parameters reduce ambiguity and allow the model to route user queries to the correct function with near-perfect precision. The key takeaway is that functions built on clear, explicit, and unique parameters deliver the highest accuracy, highlighting the importance of minimizing interpretive ambiguity when designing LLM-powered analytical systems. This takeaway mirrors the findings of the studies [47], [48], which state that clarity in the function definitions (including parameter schemas) enables the LLM to effectively plan which functions to call.

When comparing the performance of the LLM (GPT-4o) and the SLM (GPT-4o-mini) based on prompt specificity, both models achieved perfect accuracy on spatial, classification, and constraint-based analysis functions due to their explicit and unambiguous parameterization. These queries depend on clearly defined inputs (e.g., coordinates, border states, or comparison operators) that provide strong cues for GenAI to accurately interpret user intent. This finding suggests that when function parameters are explicit, complex queries with multiple details do not reduce routing accuracy. This finding is backed by the study [49], which shows that even a minor change in an argument can lead to significantly different downstream results.

As the function space becomes more densely populated with semantically overlapping functions (tools), routing accuracy degrades due to increased ambiguity at the intent–function boundary. This is also evident in our case, as there is frequent misrouting between closely related functions such as multi-indicator performance and top/bottom districts, as well as state multi-indicator and state-wise indicator extremes, which share parameters (e.g., indicator sets, performance type, geographic scope) and partially overlapping intents. This behavior is consistent with prior findings in tool-use and function-calling evaluations [50], [51]. Across models, these confusions persist even for easy and medium queries. This indicates that semantic overlaps also drive routing errors, rather than query difficulty alone.

Compared with the LLM, the SLM struggled the most with multi-indicator functions, such as the “Multi Indicator Performance” and “State Multi Indicator Performance” queries. These tasks are challenging because they require handling multiple operations simultaneously. The key takeaway is that GPT-4o-mini requires more explicit parameterization to reduce errors, whereas GPT-4o’s larger size and stronger reasoning capacity not only improve overall accuracy but also enable it to handle vague or underspecified queries more reliably [52], [53]. This finding is consistent with empirical scaling laws [41], which suggest that larger models are better at interpreting incomplete prompts and mapping them to the correct functions, while smaller models often require queries to be phrased with precise parameters.

In what follows, we outline several recommendations for future developers of GenAI-powered dashboards based on our performance evaluation results. First, the choice between an SLM and an LLM should be guided by both the system’s intended use case and the need for fine-tuning. If no fine-tuning is applied, SLMs represent the most cost-effective option for systems designed around simple, unambiguous, and explicit queries, as they perform at a level comparable to LLMs. This is supported by our findings on spatial, classification, and constraint-based functions, where queries are explicit and unambiguous, and both models achieved high accuracy. Second, LLMs are better suited for chatbots that must handle a broader spectrum of tasks, including complex, multi-parameter queries where SLMs struggled. Our results show that LLMs are more capable of addressing such cases, although this advantage comes with the tradeoff of substantially higher operational costs compared to SLMs.

Third, in real-world deployment, fine-tuning is often necessary to align the model more closely with user data and query patterns [41], [54]. Since both SLMs and LLMs perform better when queries are explicit and parameter-rich, fine-tuning should focus on enabling the system to interpret vague or underspecified inputs and map them to precise, actionable queries. For instance, a vague request such as “Is women’s health improving in Bihar?” should be translated into a more specific query like “What is the current prevalence of anemia among women in Bihar compared to previous years?”. Moreover, when users provide vague queries, the chatbot could employ a step-by-step reasoning approach to engage in more intelligent conversations and better capture the user’s implicit intent.

Despite the strengths of the proposed GenAI-powered chatbot’s architecture and workflow, several limitations should be acknowledged. First, the evaluation was restricted to two models, GPT-4o-mini and GPT-4o. Although these models demonstrate strong performance, they are proprietary and involve operational costs, which may limit their feasibility for real-world applications. Moreover, users have limited control over their behavior and functionality. By contrast, open-source LLMs release model weights and allow full customization of training data, tuning methods, and deployment environments. Leveraging such models would not only reduce usage costs but also provide greater flexibility for adapting the system to domain-specific requirements.

Second, although the chatbot is designed to make data more accessible for both general audiences and policymakers, user studies on its real-world usability and effectiveness have not yet been conducted. Because the system was developed to address the shortcomings and navigation difficulties of dashboards, future research should empirically evaluate the user experience in practice. In addition to general usability, it will be important to gather feedback from policymakers on the effectiveness of the included functions for decision-making.

Third, the current implementation relies on hardcoded Python functions, which limit the range of supported queries. The predefined set of queries was informed by the local knowledge and contextual understanding gained from developing the IPI dashboard, allowing coverage of a reasonable range of plausible use cases. However, the chatbot can only respond to questions that are mapped to these predefined functions. Despite this limitation, the proof-of-concept makes a significant contribution to the literature by demonstrating the successful integration of GenAI with interactive dashboards. Expanding the system to include fine-tuning for automated code generation could substantially increase the range of queries the chatbot is able to process, making it more adaptable and robust in practice. Prior studies [17], [55] have shown that fine-tuned LLMs can achieve near-perfect accuracy, significantly outperforming baseline models while reducing execution errors.

Although the system currently operates on public, aggregate indicators, exposing an LLM-based query interface over policy-relevant data necessitates careful consideration of security and safe use. This prototype incorporates baseline safeguards, such as constrained function execution and structured backend queries, that reduce the risk of prompt injection and unintended operations. However, a production-ready deployment would require stronger access controls to regulate who can query which data and functions. Additional measures, including rate limiting, multilingual support, and query complexity constraints, are needed to prevent denial-of-service-style queries and ensure sustainable scalability (further discussion included in the supplementary materials).

## 5. Conclusion

This proof-of-concept study demonstrated how LLM-powered chatbots can complement dashboards by broadening accessibility and enabling natural language interaction with complex policy datasets. By evaluating GenAI models (GPT-4o and GPT-4o-mini) across diverse real-world queries that varied by type, specificity, and difficulty, we highlighted the strengths of explicit parameter-driven functions as well as the challenges posed by overlapping categories. The results underscore the potential of GenAI-powered interactive dashboards to enable language-driven interaction and expand accessibility for diverse user groups, regardless of technological literacy. This approach provides a pathway to identify vulnerable areas and to close gaps between data and insights that often persist in traditional dashboards. Although this proof-of-concept study focused on the IPI dataset, the proposed framework and pipeline can be adapted to other datasets and scaled for broader applications. By improving public literacy in these domains, the study points to the potential for democratizing participation in evidence-based policymaking and strengthening accountability mechanisms. Future work should expand model options, broaden query coverage, and incorporate user studies to ensure practical utility, ultimately bridging the gap between data and insights.

## Supplementary Material

sup_info

The Supplementary Materials can be accessed at the following link: https://www.tandfonline.com/doi/suppl/10.1080/00330124.2026.2667385?scroll=top
