# Supplementary material for: Expanding Public Access to Understanding Data: A Case Study of Leveraging Generative AI for India Policy Insights Dashboard
Source: Prof Geogr. Author manuscript; Available in PMC 2026 Jul 29. (PMC13411144; doi:10.1080/00330124.2026.2667385)
Supplement: sup_info [file NIHMS2193496-supplement-sup_info.pdf]

Technical details of the chatbot architecture

Please find the github link for the code base in the following link: **cga-harvard/GenAI-for-IPI: Chatbot for Indian-Policy-Insights**

Our chatbot is organized into three layers, each described below.

Layer 1: Web and Presentation Tier (Front End)

Figure 1. Frontend operation flow for the user query, "Give me districts within 50 km of Delhi and their hypertension values." Upon query submission (Steps 1–2), the frontend parses the user input to extract key parameters such as the center location, radius, and indicator intent, maintains session context, and routes the request appropriately. Based on the parsed intent, the system determines that a spatial query requiring both map and chart visualizations is needed (Step 3). The query and session context are then forwarded to the backend API (Step 4). After receiving the processed response, the frontend updates the UI state and renders district boundaries on an interactive map (Step 9) and indicator values using Chart.js-based comparative charts (Step 10). Users can interact with the visualizations through pan, zoom, and tooltip inspection (Steps 11–12), and optionally export the rendered map or charts as images using HTML2Canvas (Steps 13–14).

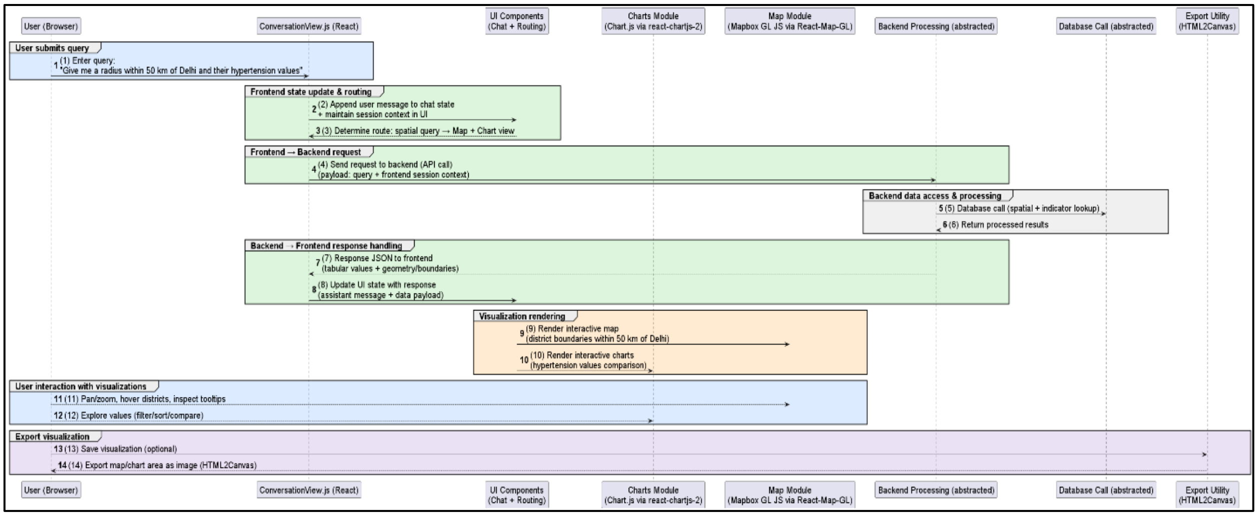

The user interface is built with React.js (v18.2.0) (Meta Platforms Inc. 2025), a JavaScript library for developing reusable user interface components. ConversationView.js serves as the main React component in our system, managing chat interactions and visualization routing. The frontend also incorporates modular components designed for different data analyses.

(1) Interface: The interface provides a conversational analytics environment that allows users to interact naturally with the data. Implemented in React.js, it manages chat sessions seamlessly and ensures effective context maintenance throughout user interactions. Visualizations generated during these interactions can be saved for future reference or sharing using HTML2Canvas (von Hertzen 2025), a JavaScript library that captures any HTML element as an image.

(2) Interactive Charts: Interactive charts provide visual analytics that enhance data understanding. They are implemented using Chart.js (Downie and Chart.js Team 2025) through React-Chartjs-2 (Ayerst and react-chartjs-2 Team 2025), open-source JavaScript libraries for creating interactive charts on web pages. These charts allow users to dynamically explore data comparisons, trends, and summaries related to SDG indicators. By supporting interactive exploration, they contribute to a more engaging user experience and empower users to make data-driven decisions more

effectively.

(3) Maps: Interactive spatial analyses are delivered through dynamic maps implemented with Mapbox GL JS (Mapbox 2025) and integrated via React-Map-GL (vis.gl 2025). These JavaScript libraries enable the display of Mapbox maps. This functionality enables users to explore geographic insights in an interactive and visual manner. The map component supports detailed geographic visualizations, helping users easily identify and analyze spatial patterns relevant to data indicators.

Layer 2: Data Analysis Tier (Back End)

Figure 2. Backend workflow for the query, "Give me districts within 50 km of Delhi and their hypertension values." The request is received and validated by the FastAPI service using predefined schemas (Step 2), after which session context is loaded, and token usage is managed through history trimming and counting (Steps 3–4). The structured prompt, including session context and registered backend functions, is passed to the GPT model for intent analysis (Step 5), where the task is identified as a spatial filtering operation and key parameters: center point, radius, and indicator, are extracted. The model then issues a function call to execute the distance-based spatial query (Steps 6–7). The backend function queries the PostgreSQL/PostGIS database to retrieve district geometries and associated indicator values via spatial joins (Steps 8–9). The results are returned as structured JSON, session memory is updated, and a natural-language response with optional boundary data is generated and sent back to the frontend for visualization (Steps 10–14).

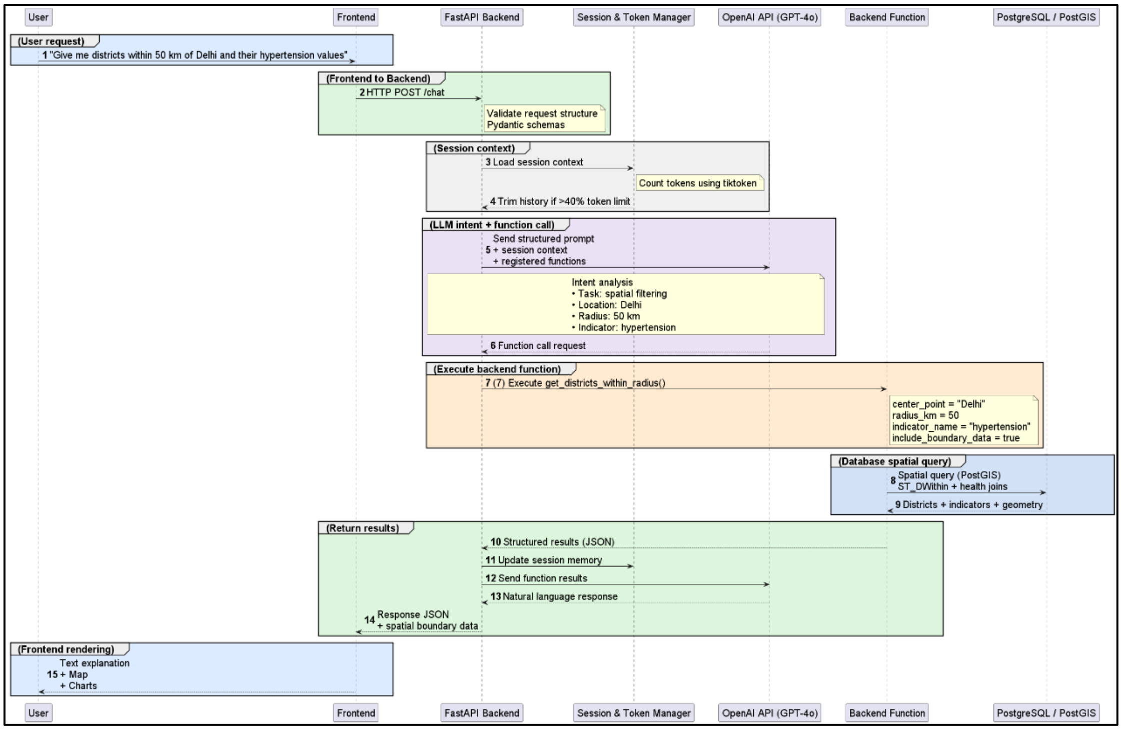

The backend architecture integrates FastAPI (Ramírez 2025) as the gateway between the frontend and backend for efficient API management. It orchestrates dynamic function-calling capabilities through OpenAI's LLM (e.g., GPT-4o) and employs a spatially enabled PostgreSQL database to support analytical processing. The main components and their roles within the backend execution workflow are described below.

(1) Request Handling via API: The backend is built with FastAPI (Ramírez 2025), a modern, high-performance Python web framework optimized for developing APIs. Its primary role is to receive HTTP requests from the frontend, validate their structure, and route them to the appropriate processing functions. FastAPI uses Pydantic (Colvin 2025) to define request and response schemas, which ensures that inputs such as user queries are automatically checked and converted into structured Python objects. This reduces errors, enforces consistency, and facilitates request processing.

In practice, when a user submits a chatbot query or requests a map or chart, FastAPI validates the request, organizes it into structured data, and forwards it to the OpenAI API. Swagger (SmartBear Software 2025) and ReDoc (Redocly 2025) FastAPI also automatically produce an interactive API documentation. They allow developers to test endpoints, inspect query payloads, and verify OpenAI-powered functions directly in the browser. By managing validation, routing, and documentation, FastAPI provides reliable and efficient communication between the frontend and backend.

(2) GenAI-based User Intent Analysis: After receiving a user request, the backend initiates GenAI-based processing to analyze the user's intent. The intent is classified into predefined categories, including task type (e.g., ranking, trend analysis, spatial filtering), spatial context (e.g., geographic scope such as districts, states, or radius-based filters), and numerical criteria (e.g., thresholds, rankings, temporal ranges). The identified intents are then structured into parameters suitable for downstream processing.

(3) Function Calling Orchestration: In the backend workflow, function calling (OpenAI 2024) enables the GenAI model (e.g., GPT-4o or GPT-4o-mini) to determine when to invoke specific backend functions rather than returning only plain text answers. During system setup, all available backend functions are registered and described to the model, including their purpose and required inputs.

When a user submits a query, GenAI interprets the intent and checks whether it aligns with one of the declared functions. If a match is found, the GenAI generates a function call request, passing the necessary parameters extracted from the query. The backend then executes the corresponding function, and the results (e.g., numbers, tables, or spatial data) are returned.

If the user's intent does not match any of the registered functions, the system bypasses function calling and produces a standard text response. This design allows the model to combine natural conversation with analytical precision, while avoiding unnecessary complexity when a function call is not required.

(4) Results Handling: Outputs from executed functions, including tabular data and spatial information, are transformed into structured formats and returned to GenAI. This enables the chatbot to incorporate accurate, context-specific analytical results from PostgreSQL/PostGIS (The PostgreSQL Global Development Group 2025b; PostGIS Development Group 2025) into its final natural language response, making the information easily understandable to users. In parallel, the raw boundary and attribute data are passed to the frontend, where they are rendered as maps and charts.

(5) Conversational Context (Session Memory) and Token Management: The backend preserves conversational coherence by storing recent exchanges in an in-memory session store, enabling the chatbot to interpret follow-up queries in context. However, retaining too much history quickly consumes tokens, which are costly and constrained by the GPT model's token limit. To address this, the system uses tiktoken (OpenAI and Jain 2025), OpenAI's official tokenizer, to count tokens before each API call. If the stored conversation exceeds 40 percent of GPT-4o's 128K token capacity (about 51K tokens), the oldest exchanges are pruned while the most recent are retained, leaving a safe buffer for model replies. This adaptive truncation ensures that long conversations remain coherent without surpassing token limits. Session memory is managed at both the frontend and backend. On the frontend, conversation history is temporarily stored so users can view the flow of their chat during a browser session. On the backend, an in-memory session store maintains the validated exchanges that are passed to the model. This dual approach combines a seamless user

experience with backend consistency.

Layer 3: Database Tier

Figure 3. Database-layer execution of the query, “Give me districts within 50 km of Delhi and their hypertension values.” The backend function opens a pooled PostgreSQL/PostGIS connection for efficient query execution (Step 1) and resolves the center point by mapping the textual location (“Delhi”) to its reference geometry and centroid (Steps 2–3). Spatial filtering is then performed using the PostGIS ST_DWithin function with GiST indexing to identify candidate districts within a 50 km radius and compute distances (Steps 4–5). The filtered districts are joined with indicator tables by matching the requested health indicator (“hypertension”), retrieving default available temporal values (e.g., 2016 and 2021), change metrics, and population counts (Steps 6–7). Finally, the database assembles the structured result set, including district identifiers, distances, indicator values, and optional boundary geometries, and returns it to the backend before releasing the connection back to the pool (Steps 8–9).

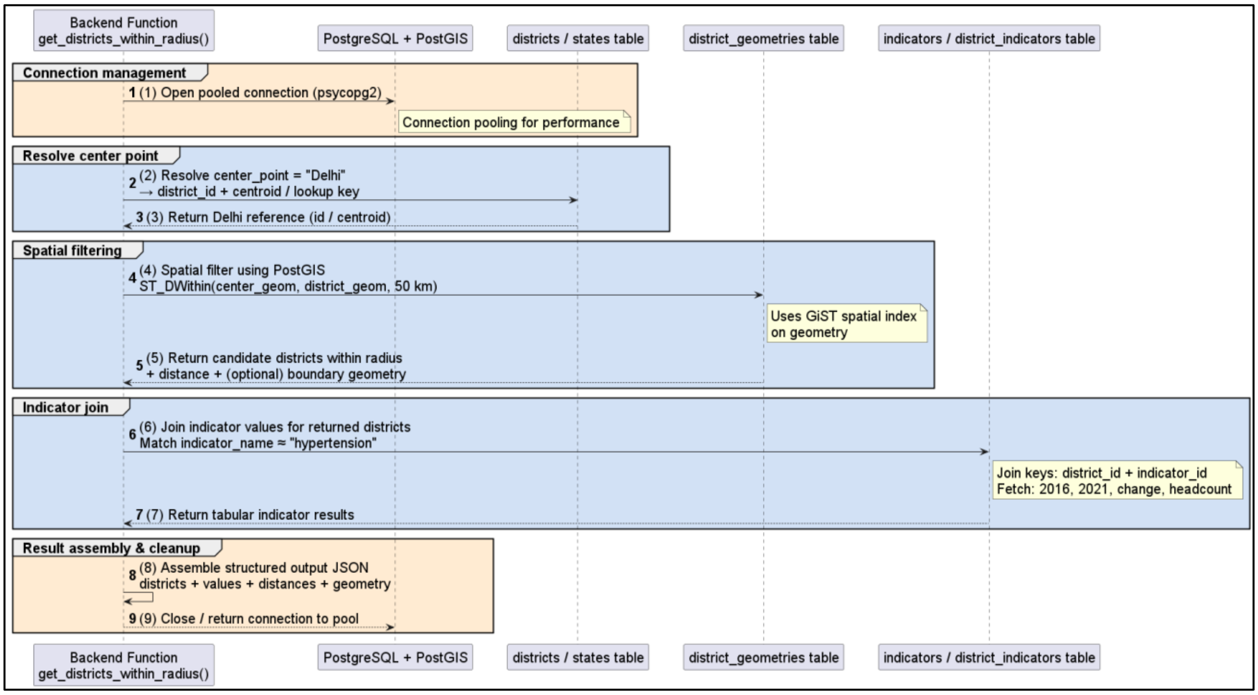

The backend uses a PostgreSQL (The PostgreSQL Global Development Group 2025b) database enhanced with PostGIS (PostGIS Development Group 2025) to manage spatially enabled datasets and analytical data. The database contains optimized tables for storing indicator values, metadata, geographic references, spatial boundaries, and user-generated visualizations. Database access and performance are improved through efficient connection pooling with psycopg2 (Di Gregorio 2025), a PostgreSQL adapter for Python that enables secure and efficient query execution.

Spatial querying is supported by PostGIS functions such as ST_Intersects and ST_DWithin, which allow rapid processing of spatial filters, neighbor lookups, and radius-based queries. To further improve speed and efficiency, the schema incorporates multiple indexes (Su and Widom 2005). These include performance indexes for ranking and filtering operations, spatial indexes using GiST (The PostgreSQL Global Development Group 2025a) for geometries and centroid columns to enhance spatial query performance, and specialized indexes optimized for filtering by state, classification, and annual changes.

Response Generation Pipeline

Figure 4. Response generation pipeline for a user query. This figure illustrates the complete response generation workflow, from user query submission and session management through LLM-based planning, backend validation and execution, database querying, and final response assembly.

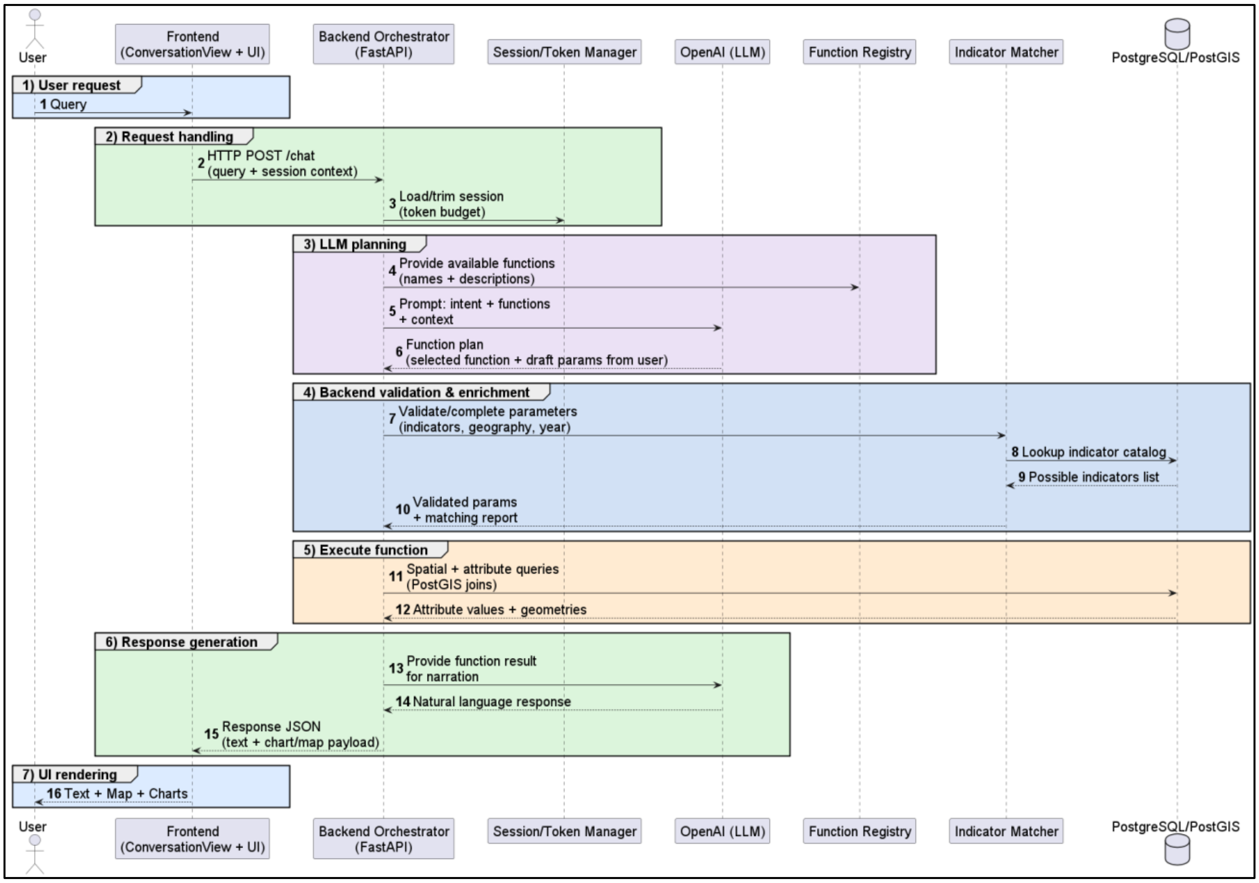

This figure presents the end-to-end response generation pipeline that transforms a user's natural-language query into an integrated textual and visual analytical response. The process begins when the user submits a query through the conversational frontend, which forwards the request along with session context to a FastAPI-based backend orchestrator. The backend manages session state and token budgets before invoking the language model for planning. During the planning stage,

the model is provided with available backend functions and their descriptions, allowing it to infer user intent and propose an executable function plan with preliminary parameters. The backend then validates and enriches these parameters by resolving geographic references and matching requested indicators against an indicator catalog present in the database. Once validated, the selected backend function executes spatial and attribute queries against the PostgreSQL/PostGIS database. The resulting data—comprising both attribute values and spatial geometries—are returned to the language model to support natural-language response generation. Finally, the backend assembles a structured response containing text, chart data, and map-ready geometries, which is sent to the frontend for rendering interactive visualizations.

Indicator Matching and Parameter Resolution

Figure 5. Indicator extraction, normalization, and matching pipeline. This figure illustrates how natural-language indicator references are extracted by the LLM, normalized, and matched against a structured indicator catalog using fuzzy similarity and confidence thresholds or LLM selection to produce a validated parameter set for downstream analytical functions.

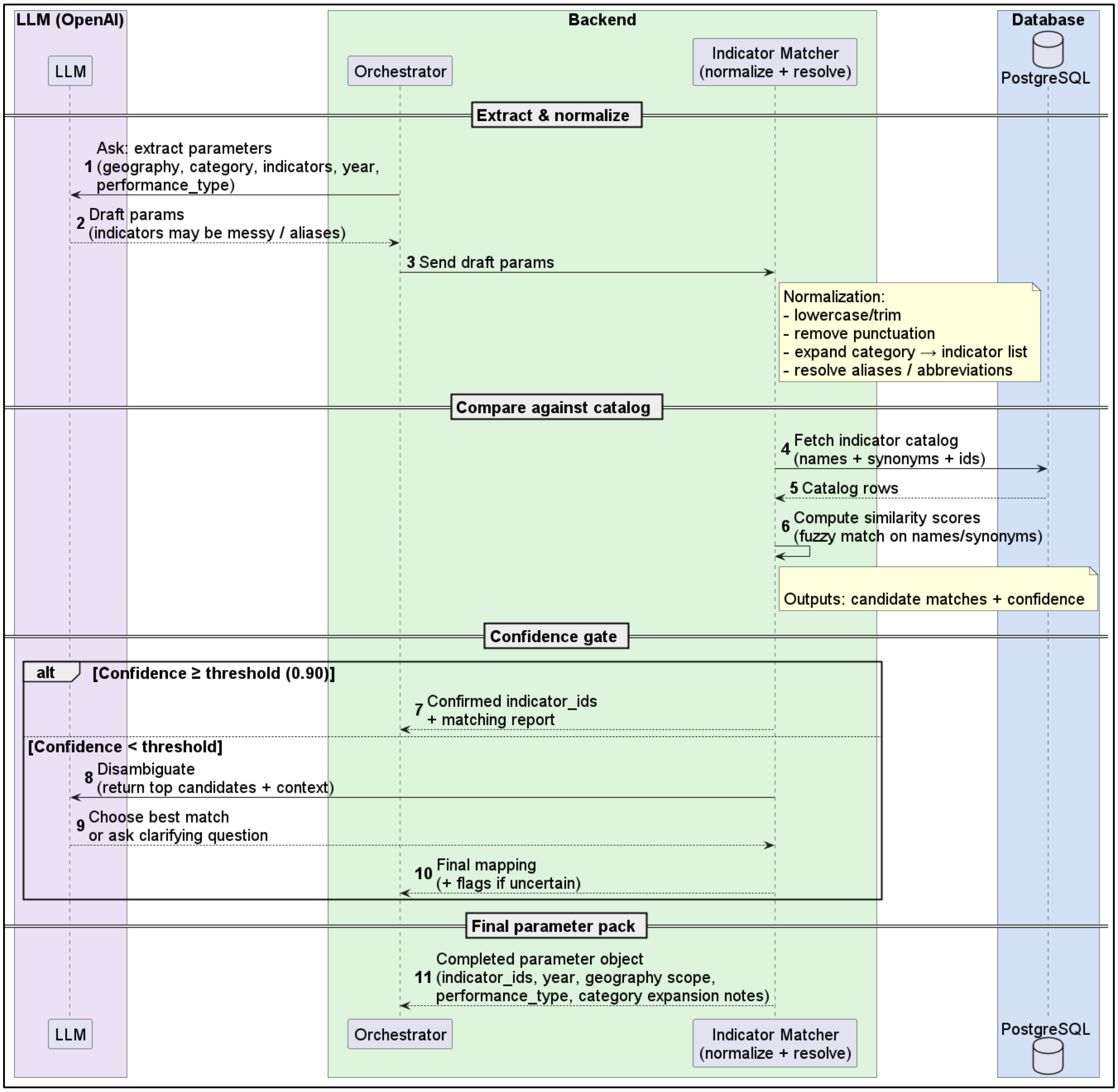

To illustrate how the system handles queries, we consider the query: **"Benchmark Mumbai, Delhi, Bengaluru Urban, and Kolkata on the nutrition category composite, showing change over 2016–2021."** Although precise, this query requires multiple intermediate steps, including category expansion into indicators, temporal comparison, composite index construction, and binding of specific district contexts.

Once the LLM identifies the intent as a multi-indicator benchmarking task, it selects the backend function get_multi_indicator_performance and extracts an initial parameter set. In this case, the extracted parameters include a list of district names (Mumbai, Delhi, Bengaluru Urban, Kolkata), a category name (nutrition), a temporal scope (2016–2021), and an implicit performance type (specific). At this stage, indicator references are not explicit; instead, the category name serves as a higher-level abstraction.

The indicator matching subsystem resolves this abstraction into a concrete set of indicators using a two-stage process. First, the category name is normalized, lowercased, trimmed, and stripped of punctuation to handle lexical variation. The normalized category (nutrition) is then matched against the indicator catalog stored in the database, which maintains a mapping between indicator IDs, indicator names, and indicator categories.

Here, the get_indicators _by_category helper Python function queries the indicators table to retrieve all indicators associated with the nutrition category (e.g., clinical nutrition, dietary intake, anthropometric indicators). This expansion step is critical: a single user- specified category is transformed into a structured indicator list with stable internal identifiers (indicator_ id) and metadata such as indicator direction (higher-is-better vs. lower-is-better).

To ensure robustness, the matching process incorporates fuzzy similarity checks and confidence thresholds. If ambiguity arises (e.g., overlapping categories or weak matches), the system can either flag uncertainty or request clarification. In this example, the category match exceeds the confidence threshold, resulting in a confirmed indicator set that is passed forward as part of the finalized parameter object. This explicit resolution step exposes the otherwise implicit reasoning between a high-level user concept ("nutrition") and the low-level indicators required for computation.

Database Fetching, Normalization, and Composite Computation

Figure 6. Execution flow of the multi-indicator performance function. This figure shows data retrieval, normalization, composite score computation, and response assembly for multi-indicator benchmarking queries.

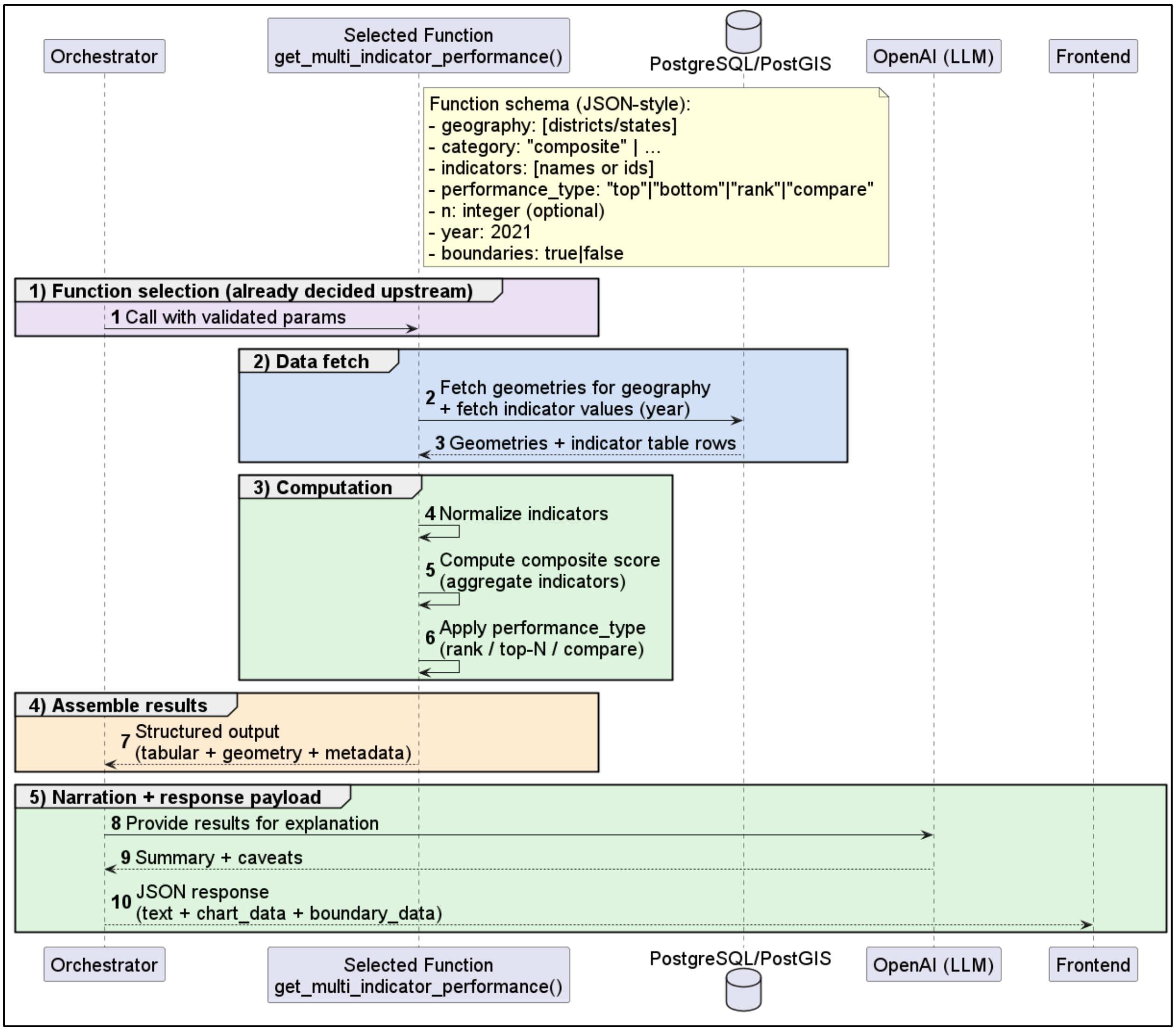

After indicator resolution, the backend proceeds to database execution and analytical computation. The selected indicator IDs are used to construct a single, comprehensive query that retrieves all required data for both normalization and comparison. Specifically, the system queries district-level

prevalence values for the selected indicators across both 2016 and 2021.

Importantly, the query is not restricted to only the four requested districts. Instead, it retrieves data for all districts nationwide that contain the selected indicators. This design choice enables global min–max normalization, ensuring that composite scores are comparable across space and time rather than being relative only to the queried subset.

The retrieved data are then passed to multi_indicator_performance function, which implements a four-step analytical pipeline:

1. Min–Max Normalization across all districts and both years.
2. Direction Alignment, inverting indicators marked as lower_is_better.
3. Composite Index Construction, computed as the average of normalized indicator values.
4. Change Analysis, calculating absolute and relative changes between 2016 and 2021.

Once composite scores are computed for all districts, the system applies the user-specified performance type. In this query, performance_type="specific" triggers a filtering step that retains only the four named districts, using fuzzy matching to account for naming variations (e.g., “Delhi” vs. “NCT of Delhi”). This sequencing of computing globally and filtering locally ensures analytical validity while preserving user intent.

Finally, district geometries are fetched in a separate step using the resolved district names. The backend assembles a structured response containing composite scores, rank context, year-over-year changes, indicator metadata, and optional geometries. This output is then forwarded to the LLM for narration and to the frontend for chart and map rendering.

Response Generation Constraints and Failure Handling

The response generation layer governs how computed results are transformed into user-readable outputs and enforces practical constraints to ensure usability and interpretability. This section focuses on interface-level limits and system behavior in edge cases.

The response length is bounded by the tokenizer limits of the underlying language model. Generated outputs are constrained by a default maximum token budget (approximately 4,096 tokens), which places an upper bound on the amount of textual explanation that can be returned in a single response. This constraint prevents excessively long or unstructured outputs and ensures consistent latency.

The visualization behavior is predefined at the function and interface level. Each backend function is associated with a fixed set of supported visualization types (e.g., a single map, a comparative bar chart), which are implemented through dedicated frontend components. If a user request implies additional maps or charts beyond what a function supports, the system responds using the predefined visualization set and includes a natural-language suggestion indicating that further outputs can be generated upon user confirmation. This stepwise interaction model avoids overwhelming the interface while still supporting exploratory analysis.

All retrieved and computed results are passed to the language model in structured form, which then generates a natural-language response that summarizes the findings and contextualizes the associated visualizations. The language model does not alter the underlying data values; it serves solely as a presentation and explanation layer for user-facing responses. The separation of computation and generation reduces the attack surface for prompt injection, as user inputs cannot directly influence database queries or numerical results beyond predefined function parameters.

The system also incorporates explicit handling for ambiguous, unsupported, or potentially unsafe queries. When user input cannot be reliably mapped to system capabilities, internal intent assessment is used to detect ambiguity. If uncertainty remains, the system generates a clarification request before executing any backend operation. Queries that fall outside the supported scope or imply excessive computational cost are gracefully declined with an explanatory message, rather than triggering partial or misleading outputs. These safeguards help mitigate denial of service style interactions and ensure that only well-defined, supported operations are executed.

The system supports multilingual interaction by relying on the language model's (OpenAI) native multilingual capabilities for query interpretation and response generation. In the current prototype, this enables basic understanding of user queries expressed in multiple languages, provided that the intent and parameters can be reliably mapped to supported backend functions. However, a production-ready deployment would require systematic evaluation across languages and finetuning the model to avoid misrepresentation of the facts presented in the local dialect, if any. This is necessary for consistent translation of system-generated explanations and visual annotations across multiple languages. These enhancements are necessary to ensure equitable access, semantic consistency, and reliable performance across diverse linguistic contexts.

While the current deployment operates on public, aggregate indicators, the architecture is designed to support future access-control mechanisms. These include role-based query permissions, rate limiting, and query complexity thresholds, which together provide a foundation for responsible use as the system scales to broader audiences and richer datasets.

Function Misrouting Analysis

Figure 7 (A-D). Function misrouting analysis across models, query specificity, and difficulty. Panels (A) and (B) show the most frequent function misrouting patterns stratified by query randomness (general vs. specific) for GPT-4o-mini and GPT-4o, respectively. Panels (C) and (D) present misrouting patterns stratified by query difficulty (easy, medium, difficult) for GPT-4o-mini and GPT-4o, respectively.

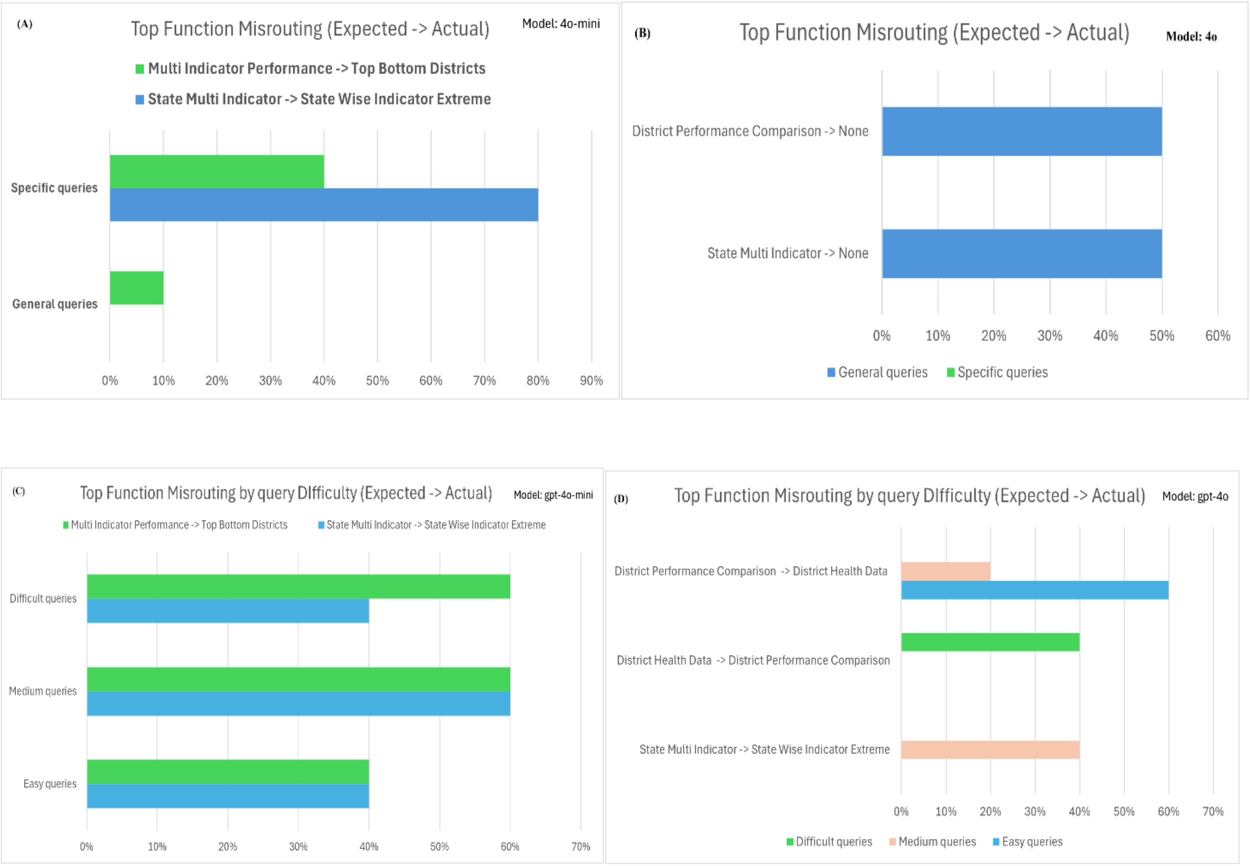

To examine how function-routing accuracy behaves under semantic overlap, we analyzed misrouting patterns across two models (GPT-4o and GPT-4o-mini), stratified by query difficulty (easy, medium, difficult) and query specificity (general vs. specific). As shown in Figures 7 A-D, the dominant misrouting cases consistently occur between pairs of functions with closely

overlapping intents and parameter schemas, such as *multi-indicator performance* versus *top/bottom districts*, and *state multi-indicator analysis* versus *state-wise indicator extremes*. These functions share core parameters (e.g., indicator sets, performance type, geographic scope) and differ primarily in aggregation logic or output framing, leading to ambiguity at the intent–function boundary even for easy and medium queries. Importantly, misrouting persists across difficulty levels and is more strongly associated with semantic proximity than with query complexity alone, suggesting that routing errors are driven by overlapping abstractions rather than insufficient query information. While the current system's limited function set allows high overall accuracy, these results indicate that scaling to a larger, more densely populated function space would likely increase routing ambiguity unless supported by additional disambiguation mechanisms (e.g., hierarchical function organization, explicit intent confirmation, or learned routing constraints). Accordingly, the present implementation is best suited for policy-analysis workflows with a curated set of high-level analytical functions, rather than unrestricted GIS-style querying over large numbers of fine-grained operations.

List of Functions

The 13 functions implemented in the system are listed along with their purpose, required parameters, optional parameters and default resolution.

Table 1. List of functions, their purpose, parameters required for the function call to be triggered and the optional parameters for the function execution.

**Function Name**	**Purpose**	**Required Parameters**	**Optional Parameters**
get_district_health_data	Get comprehensive health data for districts	district_names **or** district_name	indicator_ids, year, state_name
get_state_wise_indicator_extremes	Find best/worst districts per state	None (but indicator required)	indicator_names, indicator_name, states, year, min_districts_per_state
get_border_districts	Find districts sharing state borders	state1	state2, indicator_ids, year, include_boundary_data, include_state_comparison
get_districts_within_radius	Find districts within radius from a center point	center_point, radius_km	indicator_ids, max_districts, include_boundary_data
get_districts_by_constraints	Find districts meeting health constraints	constraints	year, states, max_districts, include_boundary_data
get_top_bottom_districts	Get top/bottom performing districts	None (but indicator required)	indicator_names, indicator_name, n_districts, performance_type, states, year, include_boundary_data
get_indicator_change_analysis	Analyze indicator changes (2016–2021)	indicator_name	analysis_level, location_name, include_boundary_data
get_district_performance_	Compare districts	district_names,	comparison_type, year,

comparison	against benchmarks	indicator_names	include_boundary_data
get_multi_indicator_performance	Multi-indicator composite performance	None	district_names, category_name, indicator_names, n_districts, year, include_boundary_data
get_state_multi_indicator_performance	State-level multi-indicator analysis	None	state_names, category_name, indicator_names, performance_type, n_districts, year, include_boundary_data
get_district_similarity_analysis	Find similar/different district patterns	None	indicator_names, category_name, analysis_type, state_names, n_districts, year, include_boundary_data
get_district_classification	Classify districts using Jenks breaks	indicator_name	state_names, year, include_boundary_data
get_district_classification_change	Classify districts by change patterns	indicator_name	state_names, include_boundary_data

Table 2. List of default value resolution of the functions if a parameter is not explicitly mentioned.

**Function Name**	**Default Parameter value resolution**
get_district_health_data	year = 2021
get_state_wise_indicator_extremes	year = 2021, min_districts_per_state = 3
get_border_districts	year = 2021, include_boundary_data = True, include_state_comparison = True

get_districts_within_radius	max_districts = 50, include_boundary_data = True, year=2021
get_districts_by_constraints	year = 2021, max_districts = 100, include_boundary_data = True
get_top_bottom_districts	n_districts = 10, performance_type = "top", year = 2021, include_boundary_data = True
get_indicator_change_analysis	analysis_level = "country", include_boundary_data = True
get_district_performance_comparison	comparison_type = "national", year = 2021, include_boundary_data = True
get_multi_indicator_performance	n_districts = 10, year = 2021, include_boundary_data = True
get_state_multi_indicator_performance	performance_type = "top", n_districts = 5, year = 2021, include_boundary_data = True
get_district_similarity_analysis	analysis_type = "similar", n_districts = 20, year = 2021, include_boundary_data = True
get_district_classification	year = 2021, include_boundary_data = True
get_district_classification_change	include_boundary_data = True

Function Classification

The 13 functions implemented in the system can be broadly classified into following categories:

(1) Spatial function queries reveal geographic patterns and location-based disparities. They help users understand how their district compares with neighboring or border districts, while also supporting policymakers in designing geographically targeted interventions, facilitating cross-border coordination, and setting priorities at the state or district level.

(2) Temporal and comparative change analysis queries track progress over time and benchmark performance. They allow the users to see whether conditions in their district are improving or worsening (e.g., vaccination coverage increasing or declining). For policymakers, these functions are essential for monitoring progress toward targets, evaluating program effectiveness, and justifying policy adjustments.

(3) Composite or multi-indicator analysis queries combine multiple measures into simplified indices or rankings. They enable both users to compare, rank, and prioritize performance across multiple indicators simultaneously.

(4) Classification and similarity analysis queries group districts based on shared characteristics to highlight patterns. For the user, these functions help explain how their district is classified (e.g., high risk, low risk, moderate performance) relative to others. For policymakers, they facilitate the clustering of similar regions to support uniform interventions, the scaling of best practices, and the targeting of specialized policies.

(5) Constraint-based analysis queries filter results by applying specific thresholds. For the user, these functions show whether their district meets defined standards (e.g., maternal care $\geq$ 80

percent and child ≤ 20 percent). For policymakers, they help identify priority districts that cross critical thresholds, ensuring more efficient targeting of funds and programs.

Comprehensive list of testing queries

Table 3. Table showing the function description and analysis category

**Name of the function**	**Description**	**Analysis Category**
Get District Health Data	Get comprehensive health indicator data (single or multiple districts/indicators) with trends, comparisons, and boundary data.	Temporal and comparative change
Get State Wise Indicator Extremes	Find the best and worst performing districts for given indicators within each state.	Spatial
Get Border Districts	Identify and analyze health performance of districts bordering one or more states.	Spatial
Get Districts Within Radius	Retrieve health indicator data for all districts within a specified distance from a center point.	Spatial
Get Districts By Constraints	Find districts that meet multiple indicator thresholds or conditions simultaneously.	Constraint based
Get Top Bottom Districts	Return top, bottom, or both performing districts for specific indicators, considering indicator direction.	Temporal and comparative change
Get Indicator Change	Analyze how indicator values changed between	Temporal and

Analysis	2016 and 2021 at country, state, or district level.	comparative change
Get District Performance Comparison	Compare multiple districts across multiple indicators against national or state benchmarks.	Temporal and comparative change
Get Multi Indicator Performance	Create a composite index across multiple indicators to assess overall or category-based district performance.	Composite or multi-indicator
Get State Multi Indicator Performance	Compare states' multi-indicator performance and highlight their top/bottom districts.	Composite or multi-indicator
Get District Similarity Analysis	Identify districts with similar or contrasting performance patterns across indicators.	Classification and Similarity
Get District Classification	Classify districts into four performance tiers for a chosen indicator using Jenks natural breaks.	Classification and Similarity
Get District Classification Change	Classify districts by improvement or decline (2016–2021) for a specific indicator using Jenks breaks.	Classification and Similarity

Table 4. Table showing queries tested for prompt specificity (General/Specific)

**Query**	**Randomness**	**Expected function**
Return 2016 and 2021 values for diabetes prevalence and full immunization coverage for Mumbai, including prevalence change and indicator direction.	Specific	Get District Health Data
For Bengaluru Urban and Bengaluru Rural, provide 2021 values for institutional delivery rate, anemia among women, and under-five stunting, with a concise comparative note.	Specific	Get District Health Data
Retrieve 2016 vs 2021 data, modern contraceptive use, and neonatal mortality rate for Mysuru	Specific	Get District Health Data
Report 2021 values for hypertension prevalence and tobacco use prevalence for Delhi, and state whether higher values are better or worse.	Specific	Get District Health Data
For Chennai, provide 2016 and 2021 values for exclusive breastfeeding (0–6 months) and low birth-weight prevalence, plus the net change.	Specific	Get District Health Data
Compare Jaipur and Ajmer for 2021 on TB prevalence, diabetes prevalence, and BP control among known hypertensives, indicating which district performs better per indicator.	Specific	Get District Health Data
For Kolkata , West Bengal, return all maternal health coverage indicators for 2016 and 2021, and summarize the trend.	Specific	Get District Health Data

Provide 2021 values for full immunization, MR vaccination, and DPT3 coverage for Lucknow, Kanpur Nagar, and Varanasi with a brief comparison.	Specific	Get District Health Data
For Thiruvananthapuram and Kollam (state: Kerala), return 2016 vs 2021 values and prevalence change for anemia in pregnant women and adult overweight/obesity.	Specific	Get District Health Data
Provide 2021 values and headcount_2021 for under-five mortality rate and stunting prevalence in Ranchi.	Specific	Get District Health Data
Provide a current health indicators overview for Surat.	General	Get District Health Data
Compare headline health metrics for Lucknow and Kanpur Nagar.	General	Get District Health Data
Summarize maternal–child indicators for Patna, noting recent trends.	General	Get District Health Data
Give a high-level indicators brief for Ernakulam and Idukki.	General	Get District Health Data
Outline non-communicable disease signals for Indore.	General	Get District Health Data
Produce an indicators snapshot for Bengaluru Urban.	General	Get District Health Data
Contrast coverage and prevalence for Jaipur and Jodhpur.	General	Get District Health Data
Provide a holistic indicators summary for Kamrup Metropolitan.	General	Get District

		Health Data
List key metrics for Varanasi with brief trend notes.	General	Get District Health Data
Create an at-a-glance comparison for Coimbatore and Tiruppur.	General	Get District Health Data
Return 2021 state-wise extremes for full immunization coverage and include trend.	Specific	Get State Wise Indicator Extremes
Provide 2016 state-wise extremes for diabetes prevalence, exclude states with fewer than five districts.	Specific	Get State Wise Indicator Extremes
Report 2021 extremes per state for anemia among pregnant women and hypertension prevalence, with trend.	Specific	Get State Wise Indicator Extremes
Give 2021 state-wise best and worst districts for institutional delivery rate for Maharashtra and Gujarat.	Specific	Get State Wise Indicator Extremes
Produce 2016 and 2021 extremes per state for under-five mortality rate with trend enabled.	Specific	Get State Wise Indicator Extremes
Return 2021 state-wise extremes for stunting and wasting among under-fives.	Specific	Get State Wise Indicator Extremes

Provide 2021 extremes per state for measles-rubella vaccination, minimum three districts per state.	Specific	Get State Wise Indicator Extremes
Show 2016 state-wise extremes for tobacco use among adults; do not include trend.	Specific	Get State Wise Indicator Extremes
Report 2021 extremes per state for antenatal care four or more visits and postnatal care within 48 hours.	Specific	Get State Wise Indicator Extremes
Provide 2021 state-wise extremes for low birth weight and exclusive breastfeeding for Kerala and Tamil Nadu.	Specific	Get State Wise Indicator Extremes
Who tops and who trails on child vaccination in every state?	General	Get State Wise Indicator Extremes
Show me best vs worst districts for skilled birth delivery across India.	General	Get State Wise Indicator Extremes
Where do states see the biggest gaps in malnutrition?	General	Get State Wise Indicator Extremes
Give a quick list of highest and lowest anaemia rates by state.	General	Get State Wise Indicator

		Extremes
Which districts lead on literacy inside each state?	General	Get State Wise Indicator Extremes
State-wise winners and losers for under-five mortality.	General	Get State Wise Indicator Extremes
Highlight districts that lag behind on clean cooking fuel within their states.	General	Get State Wise Indicator Extremes
Best and worst on household electricity access across all states?	General	Get State Wise Indicator Extremes
Who’s at the top and bottom for contraceptive use state by state?	General	Get State Wise Indicator Extremes
Show extremes for safe drinking-water coverage in every state.	General	Get State Wise Indicator Extremes
For 2021, analyse vaccination coverage and institutional delivery rate in districts bordering Maharashtra, benchmarking each district against the Maharashtra average.	Specific	Get Border Districts
Examine diabetes prevalence and hypertension prevalence in	Specific	Get Border

districts along the Karnataka–Tamil Nadu border, comparing each district with the average of its own state.		Districts
Report anemia among women and under-five stunting in districts bordering Gujarat for 2016.	Specific	Get Border Districts
Assess tuberculosis prevalence and adult tobacco use in districts on the Assam–West Bengal border, contrasting district outcomes with the relevant state mean.	Specific	Get Border Districts
Evaluate modern contraceptive use and postnatal care within 48 hours across districts adjoining Rajasthan, situating findings relative to the Rajasthan average for 2016 data.	Specific	Get Border Districts
For 2021, provide a comprehensive profile across all available indicators for districts bordering Bihar, indicating how each compares with the Bihar statewide average.	Specific	Get Border Districts
Summarize neonatal mortality rate and low birth weight in districts along the Uttar Pradesh–Madhya Pradesh border.	Specific	Get Border Districts
Present full immunization coverage and measles–rubella vaccination for districts bordering Punjab, comparing district values with the Punjab average.	Specific	Get Border Districts
Analyze antenatal care four or more visits and exclusive breastfeeding in districts situated on the Odisha–Chhattisgarh border.	Specific	Get Border Districts
In 2021, assess diabetes prevalence and hypertension control among known cases in districts adjacent to Delhi, comparing	Specific	Get Border Districts

district performance with the Delhi average.		
Which districts sit right on the Maharashtra border and how are they doing?	General	Get Border Districts
Show me border areas around Karnataka on notable health gaps.	General	Get Border Districts
Who leads on average performance along the Tamil Nadu edge?	General	Get Border Districts
Compare Gujarat's neighboring districts for health.	General	Get Border Districts
Where do states meet Rajasthan, and who's doing well?	General	Get Border Districts
Highlight border districts next to Bihar that are lagging.	General	Get Border Districts
Give me nutrition performance for districts touching Odisha.	General	Get Border Districts
Find districts on the Assam border and rate them.	General	Get Border Districts
How are boundary districts around Uttar Pradesh performing?	General	Get Border Districts
Any insights on development patterns along the Kerala border?	General	Get Border Districts
From Delhi, within 150 km, return diabetes prevalence with 2016 and 2021 values, change over time, headcount context, and	Specific	Get Districts Within Radius

distances; limit to 40 districts.		
Using 28.6139,77.2090 as the center and a 200 km radius, report full immunization coverage with 2016 and 2021 values, change, headcount, and distance ranking.	Specific	Get Districts Within Radius
From Bhopal, within 100 km, provide neonatal mortality rate with baseline 2016, 2021 level, calculated change, headcount, and distances.	Specific	Get Districts Within Radius
From Kolkata, return institutional delivery rate with 2016–2021 comparison, change, headcount, and distance ordering, up to 50 districts.	Specific	Get Districts Within Radius
From Jaipur, within 150 km, report stunting among under-fives with 2016 and 2021 values, change, headcount context, and distances.	Specific	Get Districts Within Radius
Using 19.0760,72.8777 as the center and a 300 km radius, provide tuberculosis prevalence with 2016–2021 comparison, change, headcount, and distance ranking.	Specific	Get Districts Within Radius
From Hyderabad, return modern contraceptive use with 2016 and 2021 values, change over time, headcount, and distances.	Specific	Get Districts Within Radius
Using 9.9312,76.2673 as the center and within some radius, report anemia among women with 2016–2021 values, change, headcount, and distance ordering.	Specific	Get Districts Within Radius
From Ahmedabad, within 200 km, provide blood pressure control among known hypertensives with 2016 and 2021 values, change,	Specific	Get Districts Within Radius

headcount, and distances.		
From Lucknow, within 150 km, return all indicators with 2016 baselines, 2021 levels, calculated change, headcount context, and distance ranking; cap at 45 districts.	Specific	Get Districts Within Radius
Show districts near Delhi that score high on education.	General	Get Districts Within Radius
Which places within a short drive of Mumbai have poor health outcomes?	General	Get Districts Within Radius
List nearby districts around Bengaluru and their mortality data.	General	Get Districts Within Radius
Who’s doing best on poverty reduction near Kolkata?	General	Get Districts Within Radius
Find all districts close to Chennai with good nutrition values	General	Get Districts Within Radius
Give me a map of areas within reach of Jaipur for family planning goals.	General	Get Districts Within Radius
Any high-performing districts within 100 km of Hyderabad?	General	Get Districts Within Radius
How are regions around Lucknow faring on gender equality?	General	Get Districts Within Radius
Show poverty levels in districts not far from Patna.	General	Get Districts Within Radius
Compare development indicators for places surrounding	General	Get Districts

Ahmedabad.		Within Radius
For 2021, list districts where diabetes prevalence > 15 and full immunization coverage < 70.	Specific	Get Districts By Constraints
Identify districts with under-five stunting ≥ 30 and wasting ≥ 15.	Specific	Get Districts By Constraints
Return districts where institutional delivery rate ≤ 60 and postnatal care within 48 hours ≤ 50.	Specific	Get Districts By Constraints
Find districts with anemia among women ≥ 55 and modern contraceptive use ≤ 45.	Specific	Get Districts By Constraints
For 2021, within Maharashtra and Gujarat, show districts where neonatal mortality rate ≥ 25 and ANC four or more visits ≤ 60.	Specific	Get Districts By Constraints
Identify districts with hypertension prevalence ≥ 25 and BP control among known hypertensives ≤ 50, in 2021.	Specific	Get Districts By Constraints
Return districts with diarrhea prevalence among under-fives ≥ 10 and exclusive breastfeeding ≤ 40.	Specific	Get Districts By Constraints
For 2021, show districts meeting vaccination coverage ≥ 90 and institutional delivery rate ≥ 95 and neonatal mortality rate ≤ 15.	Specific	Get Districts By Constraints
In Kerala and Tamil Nadu, list districts with low birth weight ≥ 20 and stunting ≥ 25.	Specific	Get Districts By Constraints
Provide up to 150 districts where adult obesity ≥ 30 and diabetes prevalence ≥ 12 and hypertension prevalence ≥ 25.	Specific	Get Districts By Constraints
Identify districts with simultaneous nutritional risk and low immunization performance.	General	Get Districts By Constraints

Find districts exhibiting high noncommunicable disease burden alongside weak maternal care coverage.	General	Get Districts By Constraints
List districts that meet multiple adverse child health conditions within a single year.	General	Get Districts By Constraints
Surface districts combining elevated infectious disease prevalence with low primary care utilization.	General	Get Districts By Constraints
Highlight districts that satisfy strong maternal–child coverage while maintaining controlled chronic disease levels.	General	Get Districts By Constraints
Retrieve districts facing overlapping anemia and stunting challenges.	General	Get Districts By Constraints
Determine districts with high neonatal mortality alongside limited antenatal care access.	General	Get Districts By Constraints
Flag districts with low institutional delivery and low postnatal care coverage.	General	Get Districts By Constraints
Find districts with high tobacco use and poor hypertension control.	General	Get Districts By Constraints
Identify districts that meet stringent targets for vaccination, ANC, and PNC simultaneously.	General	Get Districts By Constraints
Provide the top 10 districts for full immunization coverage in 2021.	Specific	Get Top Bottom Districts
List the bottom 5 districts for diabetes prevalence in 2021.	Specific	Get Top Bottom

		Districts
Report the top 12 and bottom 12 districts for under-five stunting in 2021.	Specific	Get Top Bottom Districts
Return the top 8 districts for institutional delivery rate in 2016 within Tamil Nadu and Kerala.	Specific	Get Top Bottom Districts
Identify the top 15 districts in 2021 by composite performance across diabetes and hypertension.	Specific	Get Top Bottom Districts
List the bottom 15 districts in 2021 by composite performance across tuberculosis prevalence and tobacco use.	Specific	Get Top Bottom Districts
Provide the top 10 districts in 2021 for antenatal care four or more visits and postnatal care within 48 hours across Maharashtra, Gujarat, and Madhya Pradesh.	Specific	Get Top Bottom Districts
Return the bottom 10 districts in 2016 for measles–rubella vaccination and DPT3 coverage within Uttar Pradesh and Bihar.	Specific	Get Top Bottom Districts
List the top 20 districts in 2021 by composite performance across exclusive breastfeeding and low birth weight.	Specific	Get Top Bottom Districts
Provide the top 10 and bottom 10 districts across all health	Specific	Get Top

indicators in 2021.		Bottom Districts
Show the top districts nationally for vaccination coverage.	General	Get Top Bottom Districts
Identify the bottom districts nationally for diabetes prevalence.	General	Get Top Bottom Districts
Report top performers for maternal health coverage.	General	Get Top Bottom Districts
List top and bottom districts for child nutrition outcomes.	General	Get Top Bottom Districts
Summarize leading districts for hypertension control.	General	Get Top Bottom Districts
Highlight lagging districts for neonatal mortality.	General	Get Top Bottom Districts
Provide a ranking of districts excelling in antenatal and postnatal care.	General	Get Top Bottom Districts

Indicate the strongest districts for tuberculosis control outcomes.	General	Get Top Bottom Districts
Compare top districts for modern contraceptive use.	General	Get Top Bottom Districts
Present top and bottom districts for overall child immunization.	General	Get Top Bottom Districts
Conduct a country-level change analysis for diabetes prevalence between NFHS 4 and 5.	Specific	Get Indicator Change Analysis
Provide a state-level change analysis of vaccination coverage for Uttar Pradesh.	Specific	Get Indicator Change Analysis
Analyze changes in child stunting for Kerala at the state level from NFHS 4 to NFHS 5.	Specific	Get Indicator Change Analysis
Report a state-level change assessment of hypertension prevalence in Gujarat over NFHS4 to NFHS 5.	Specific	Get Indicator Change Analysis
Examine state-level changes in anemia among women for Bihar between NFHS4 and 5.	Specific	Get Indicator Change

		Analysis
Present a district-level change analysis for malnutrition in Mumbai from 2016 to 2021.	Specific	Get Indicator Change Analysis
Provide a district-level trend for neonatal mortality rate in Jaipur over NFHS4 to NFHS 5.	Specific	Get Indicator Change Analysis
Assess district-level changes in institutional delivery rate in Patna between NFHS4 and 5.	Specific	Get Indicator Change Analysis
Analyze state-level changes in exclusive breastfeeding for Tamil Nadu from 2016 to 2021.	Specific	Get Indicator Change Analysis
Conduct a country-level change assessment for postnatal care within 48 hours over NFHS4 to NFHS 5.	Specific	Get Indicator Change Analysis
Evaluate national changes in diabetes prevalence.	General	Get Indicator Change Analysis
Assess how vaccination coverage shifted nationally.	General	Get Indicator Change Analysis
Summarize the change in child stunting for India.	General	Get Indicator

		Change Analysis
Examine national trends in hypertension prevalence.	General	Get Indicator Change Analysis
Describe how institutional delivery rate changed nationally.	General	Get Indicator Change Analysis
Review the change in neonatal mortality rate at the country level.	General	Get Indicator Change Analysis
Assess whether anemia among women improved nationally.	General	Get Indicator Change Analysis
Summarize changes in exclusive breastfeeding at the national level.	General	Get Indicator Change Analysis
Evaluate national shifts in tuberculosis prevalence.	General	Get Indicator Change Analysis
Describe how modern contraceptive use changed in India.	General	Get Indicator Change Analysis

Compare diabetes prevalence and hypertension prevalence for Mumbai, Delhi, Bengaluru Urban, and Kolkata against national averages, and summarize performance gaps.	Specific	Get District Performance Comparison
Benchmark institutional delivery rate, antenatal care four or more visits, and postnatal care within 48 hours for Chennai, Hyderabad, Pune, and Ahmedabad against their respective state averages.	Specific	Get District Performance Comparison
Compare full immunization coverage and DPT3 coverage for Lucknow, Kanpur Nagar, Varanasi, and Prayagraj against national averages.	Specific	Get District Performance Comparison
For 2021, evaluate under-five stunting and wasting for Jaipur, Jodhpur, Kota, and Udaipur relative to state averages.	Specific	Get District Performance Comparison
Compare anemia among women and low birth weight for Patna, Gaya, Muzaffarpur, and Bhagalpur against national averages.	Specific	Get District Performance Comparison
For 2016 data, benchmark tuberculosis prevalence and tobacco use among adults for Indore, Bhopal, Gwalior, and Ujjain relative to state averages.	Specific	Get District Performance Comparison
For NFHS 4, compare modern contraceptive use and institutional delivery rate for Coimbatore, Madurai, Tiruchirappalli, and Salem against national averages.	Specific	Get District Performance Comparison
Evaluate exclusive breastfeeding and neonatal mortality rate for Thiruvananthapuram, Ernakulam, Kozhikode, and Kollam	Specific	Get District Performance

relative to state averages in 2021.		Comparison
Benchmark measles–rubella vaccination and full immunization coverage for Ahmedabad, Surat, Rajkot, and Vadodara against national averages using NFHS 4 data.	Specific	Get District Performance Comparison
For 2021, compare BP control among known hypertensives and diabetes prevalence for Ranchi, Dhanbad, Jamshedpur (East Singhbhum), and Hazaribagh versus state averages.	Specific	Get District Performance Comparison
Benchmark Guntur's across key indicators against national averages.	General	Get District Performance Comparison
Compare multiple districts in Sikkim relative to their respective state averages.	General	Get District Performance Comparison
Assess multi-district performance gaps versus national benchmarks.	General	Get District Performance Comparison
Contrast districts on selected indicators using state-level benchmarking.	General	Get District Performance Comparison
Provide a multi-district overview against national standards.	General	Get District Performance Comparison
Identify districts that consistently outperform national averages	General	Get District

across multiple indicators.		Performance Comparison
Flag districts underperforming relative to state averages among Bengaluru, Kota and Chennai.	General	Get District Performance Comparison
Summarize North Goa's performance around national benchmarks for priority indicators.	General	Get District Performance Comparison
Rank Amritsar and Kolkata by composite performance relative to national baselines.	General	Get District Performance Comparison
Quantify multi-indicator gaps between Delhi and Mumbai and their state averages.	General	Get District Performance Comparison
As of 2021, produce the national top 15 districts on the healthcare category composite index.	Specific	Get Multi Indicator Peformance
Using 2021 as the analysis year, list the national bottom 10 districts in the nutrition category.	Specific	Get Multi Indicator Peformance
In 2021, report both the top 10 and bottom 10 districts for the maternal health category.	Specific	Get Multi Indicator Peformance

Benchmark Mumbai, Delhi, Bengaluru Urban, and Kolkata on the nutrition category composite, showing change over 2016–2021.	Specific	Get Multi Indicator Peformance
For the year 2016, rank the national top 12 districts by a composite built from diabetes prevalence, hypertension prevalence, and tobacco use among adults.	Specific	Get Multi Indicator Peformance
With 2021 as the reference year, identify the national bottom 15 districts based on a composite of under-five stunting, wasting, and low birth weight.	Specific	Get Multi Indicator Peformance
Return the national top 10 districts for exclusive breastfeeding, institutional delivery rate, and antenatal care four or more visits, using 2021 values.	Specific	Get Multi Indicator Peformance
Evaluate the mortality category for 2021 and return both top 8 and bottom 8 districts by composite score.	Specific	Get Multi Indicator Peformance
Using 2021 values, compare Jaipur, Jodhpur, Kota, and Udaipur on the healthcare category composite and report relative standings.	Specific	Get Multi Indicator Peformance
Set the analysis year to 2021 and list the national top 20 districts by a composite of vaccination coverage, measles–rubella vaccination, DPT3 coverage, and postnatal care within 48 hours.	Specific	Get Multi Indicator Peformance
Identify top districts nationally by composite healthcare performance.	General	Get Multi Indicator

		Peformance
Flag districts with the weakest nutrition composite scores.	General	Get Multi Indicator Peformance
Provide a multi-indicator performance overview for Mumbai, Delhi, and Chennai.	General	Get Multi Indicator Peformance
Compare composite maternal health performance across five major urban districts.	General	Get Multi Indicator Peformance
Summarize national dispersion in mortality-related composite scores.	General	Get Multi Indicator Peformance
Highlight districts showing broad-based improvement across multiple indicators since 2016.	General	Get Multi Indicator Peformance
Rank districts by overall health composite and note prominent outliers.	General	Get Multi Indicator Peformance
Provide a representative cross-section of districts to illustrate variance in composite performance.	General	Get Multi Indicator Peformance
Identify districts that perform strongly across healthcare, nutrition,	General	Get Multi

and maternal health composites simultaneously.		Indicator Peformance
Present a national leaderboard and tail for the overall multi-indicator composite.	General	Get Multi Indicator Peformance
As of 2021, compare Maharashtra and Gujarat on the healthcare category and list each state’s top 5 districts.	Specific	Get State Multi Indicator Peformance
Using 2021 as the reference year, evaluate Kerala and Tamil Nadu for nutrition and report the lowest 5 districts in each state.	Specific	Get State Multi Indicator Peformance
For the year 2016, benchmark Uttar Pradesh and Bihar on maternal health and show both the top 3 and bottom 3 districts per state.	Specific	Get State Multi Indicator Peformance
In 2021, assess Punjab, Haryana, and Rajasthan using indicator set: full immunization coverage, DPT3 coverage, measles–rubella vaccination, and postnatal care within 48 hours; return the top 5 districts in each state.	Specific	Get State Multi Indicator Peformance
With 2021 as the analysis year, compare Assam, Meghalaya, and Nagaland on maternal health, highlighting the worst 4 districts in	Specific	Get State Multi

each state.		Indicator Peformance
Set the year to 2021 and evaluate Maharashtra and Telangana across diabetes prevalence, hypertension prevalence, and tobacco use among adults, returning the top 4 districts per state.	Specific	Get State Multi Indicator Peformance
For 2021, analyze West Bengal in the healthcare category and provide both top 5 and bottom 5 districts.	Specific	Get State Multi Indicator Peformance
Using 2016 data, compare Odisha and Chhattisgarh on mortality and list the lowest 3 districts in each state.	Specific	Get State Multi Indicator Peformance
In 2021, examine Karnataka and Andhra Pradesh for nutrition and surface the top 6 districts per state.	Specific	Get State Multi Indicator Peformance
For 2021, evaluate Rajasthan and Madhya Pradesh on nutrition and return both top 4 and bottom 4 districts in each state.	Specific	Get State Multi Indicator Peformance
Compare state-level composite performance and highlight each	General	Get State

state’s top districts.		Multi Indicator Peformance
Identify states with the strongest nutrition composites and list their leading districts.	General	Get State Multi Indicator Peformance
Summarize state maternal health composites and flag the weakest districts within each state.	General	Get State Multi Indicator Peformance
Provide a multi-state overview of healthcare composites with district benchmarks.	General	Get State Multi Indicator Peformance
Rank states by mortality-related composite scores and surface the bottom districts.	General	Get State Multi Indicator Peformance
Assess inter-state variation in composites and indicate exemplary districts.	General	Get State Multi Indicator Peformance

Outline state composites for priority domains and call out underperforming districts.	General	Get State Multi Indicator Peformance
Map state performance gaps and list districts that anchor each state's results.	General	Get State Multi Indicator Peformance
Present a cross-state composite dashboard with top and bottom districts.	General	Get State Multi Indicator Peformance
Profile state composites across domains and identify districts most in need of support.	General	Get State Multi Indicator Peformance
As of 2021, identify up to 16 districts nationwide that show similar patterns across diabetes prevalence, hypertension prevalence, tobacco use among adults.	Specific	Get District Similarity Analysis
For the year 2016, within Kerala and Tamil Nadu, surface 12 districts with different patterns across exclusive breastfeeding, low birth weight, neonatal mortality rate.	Specific	Get District Similarity Analysis
Using 2021 data, in Uttar Pradesh, Bihar, Jharkhand, find 20	Specific	Get District

districts with similar performance based on the nutrition category (auto-select four indicators).		Similarity Analysis
In 2021, across Karnataka and Maharashtra, identify 15 districts with different patterns in vaccination coverage, DPT3 coverage, measles–rubella vaccination.	Specific	Get District Similarity Analysis
Set the analysis year to 2016 and, across Odisha and Chhattisgarh, return 10 districts with similar profiles in institutional delivery rate, antenatal care four or more visits, postnatal care within 48 hours.	Specific	Get District Similarity Analysis
With 2021 as reference, nationwide, select 18 districts that are different in the maternal health category, randomly choose 5 indicators.	Specific	Get District Similarity Analysis
Evaluate 2021 results for Punjab, Haryana, Rajasthan and list 14 districts that are similar across under-five stunting, wasting, low birth weight.	Specific	Get District Similarity Analysis
Using 2016 values, identify 10 districts in Assam and West Bengal with different patterns in tuberculosis prevalence, tobacco use among adults.	Specific	Get District Similarity Analysis
For 2021, across Andhra Pradesh and Telangana, return 20 districts that are similar in the healthcare category.	Specific	Get District Similarity Analysis
In 2016, nationwide, isolate 8 districts with different patterns across modern contraceptive use, institutional delivery rate,	Specific	Get District Similarity

exclusive breastfeeding.		Analysis
Identify clusters of districts with similar nutrition performance patterns.	General	Get District Similarity Analysis
Find districts that display contrasting maternal health trajectories.	General	Get District Similarity Analysis
Surface groups of districts with comparable healthcare coverage profiles.	General	Get District Similarity Analysis
Highlight districts whose mortality indicators diverge markedly from peers.	General	Get District Similarity Analysis
Discover districts with aligned noncommunicable disease patterns.	General	Get District Similarity Analysis
Reveal districts that differ across key child health indicators.	General	Get District Similarity Analysis
Map clusters of districts sharing similar immunization outcomes.	General	Get District Similarity Analysis
Detect districts with opposing patterns in service utilization.	General	Get District

		Similarity Analysis
Group districts by resemblance across priority indicators.	General	Get District Similarity Analysis
Isolate districts exhibiting distinct multi-indicator profiles	General	Get District Similarity Analysis
For 2021, classify all districts in India by full immunization coverage.	Specific	Get District Classification
Create a 2016 classification map of diabetes prevalence for Maharashtra and Gujarat.	Specific	Get District Classification
In 2021, generate district performance categories choropleth map for under-five stunting within Uttar Pradesh, Bihar, and Jharkhand.	Specific	Get District Classification
Using 2016 data, classify districts nationwide by institutional delivery rate.	Specific	Get District Classification
For 2021, derive a classification for anemia among women across Kerala and Tamil Nadu.	Specific	Get District Classification
Classify districts by neonatal mortality rate for 2021 at the national level.	Specific	Get District Classification
For 2016, produce a classification for hypertension prevalence across Rajasthan and Madhya Pradesh.	Specific	Get District Classification

In 2021, present a district classification for exclusive breastfeeding within Andhra Pradesh and Telangana.	Specific	Get District Classification
Using 2021 values, classify districts by tuberculosis prevalence across West Bengal, Odisha, and Chhattisgarh.	Specific	Get District Classification
Provide a 2016 national classification for modern contraceptive use	Specific	Get District Classification
Classify districts nationwide by vaccination coverage performance.	General	Get District Classification
Produce a national division choropleth map of diabetes prevalence across districts.	General	Get District Classification
Generate district performance categories for under-five stunting.	General	Get District Classification
Create a nationwide classification for institutional delivery rate.	General	Get District Classification
Segment districts by anemia among women to identify performance categories.	General	Get District Classification
Classify districts by neonatal mortality rate at the national scale.	General	Get District Classification
Present a district classification for hypertension prevalence.	General	Get District Classification
Develop a nationwide map diving districts for exclusive breastfeeding data.	General	Get District Classification
Classify districts by tuberculosis prevalence to highlight	General	Get District

performance patterns.		Classification
Provide a choropleth map for modern contraceptive use across districts.	General	Get District Classification

Table 5. Table showing queries tested for prompt difficulty (Easy/Medium/Hard)

Easy	Medium	Hard	Expected function
Provide all health indicators for Surat.	For 2016, provide diabetes prevalence for Bhopal.	For 2021, compare Mumbai, Delhi, and Kolkata on diabetes, hypertension, and tobacco use among adults.	Get District Health Data
Return diabetes prevalence for Mumbai.	Compare institutional delivery rate for Ranchi and Dhanbad.	Provide 2016 values for ANC four or more visits and institutional delivery rate for Jaipur, Ajmer, and Udaipur.	Get District Health Data
Provide full immunization coverage for Jaipur.	Provide exclusive breastfeeding and low birth weight for Chennai.	In 2021, return full immunization coverage and measles–rubella vaccination for Lucknow, Kanpur Nagar, and Varanasi.	Get District Health Data
Report tuberculosis prevalence for Coimbatore.	For 2021, return neonatal mortality rate for Varanasi.	Provide 2016 data for neonatal mortality rate and low birth weight for Chennai, Madurai, and Tiruchirappalli.	Get District Health Data
Summarize all indicators for	Provide 2016 hypertension prevalence	For 2021, compare Thiruvananthapuram,	Get District Health Data

Patna.	for Indore.	Ernakulam, and Kozhikode on exclusive breastfeeding, institutional delivery rate, and ANC four or more visits.	
State-wise best and worst districts for vaccination coverage.	For 2016, provide state-wise extremes for diabetes prevalence.	For 2021, across Uttar Pradesh, Bihar, and Jharkhand, report state-wise extremes for stunting, wasting, and low birth weight with trend analysis.	Get State Wise Indicator Extremes
Identify state-wise extremes for diabetes prevalence.	Show state-wise extremes for hypertension prevalence in Maharashtra and Gujarat.	Compare Punjab, Haryana, Rajasthan, showing best and worst districts per state for full immunization coverage and measles–rubella vaccination, requiring at least four districts per state.	Get State Wise Indicator Extremes
Report top and bottom districts per state for anemia among women.	Provide state-wise extremes for anemia among women, minimum five districts per state.	For 2016, provide state-wise extremes for diabetes prevalence and hypertension prevalence across Karnataka, Andhra Pradesh, and Telangana.	Get State Wise Indicator Extremes

Show state-wise extremes for under-five stunting.	In Kerala and Tamil Nadu, report state-wise extremes for exclusive breastfeeding.	In Assam, Meghalaya, Nagaland, list best and worst districts per state for tuberculosis prevalence and tobacco use among adults in 2021, noting intra-state gaps.	Get State Wise Indicator Extremes
List best and worst districts in every state for institutional delivery rate.	For 2021, list state-wise extremes for neonatal mortality rate with a brief trend note.	For 2021, show state-wise extremes for ANC four or more visits, institutional delivery rate, and postnatal care within 48 hours across West Bengal and Odisha, enforcing a minimum of five districts per state.	Get State Wise Indicator Extremes
List districts that border Maharashtra and summarize their health performance.	For 2016, compare vaccination coverage in districts bordering Delhi with the Delhi average.	In 2021, analyze diabetes prevalence and hypertension prevalence for districts on the Karnataka–Maharashtra border, benchmarking each district against its own state average.	Get Border Districts
Identify districts adjoining	Between Karnataka and Tamil Nadu, examine	For 2016, evaluate full immunization coverage,	Get Border Districts

Karnataka and provide a brief health overview.	overall health outcomes in their shared border districts.	DPT3 coverage, and measles–rubella vaccination in districts bordering Punjab, with comparisons to the Punjab average.	
Show districts near Tamil Nadu.	Assess diabetes prevalence in districts bordering Gujarat, benchmarking against the Gujarat average.	In 2021, assess anemia among women and under-five stunting for districts along the Odisha–Chhattisgarh border and discuss cross-state contrasts.	Get Border Districts
Provide a health profile of districts surrounding Uttar Pradesh.	For 2021, review institutional delivery rate in districts adjoining Rajasthan.	For 2016, compare neonatal mortality rate and low birth weight across districts adjacent to Uttar Pradesh, situating results relative to the UP average.	Get Border Districts
Summarize information of districts close to Assam.	Compare districts around the Bihar border with the statewide average for a single priority indicator of your choice.	In 2021, examine modern contraceptive use, ANC four or more visits, and postnatal care within 48 hours for districts on the Kerala–Tamil Nadu border, with benchmark	Get Border Districts

		comparisons to each state's average.	
Within 90 km of Nagpur, return districts data for maternal health.	Using the coordinates 22.5726, 88.3639 as the centre point, list up to 28 districts within a 180-kilometre radius, report their diabetes prevalence, and omit all boundary geometry from the response.	With 19.0760, 72.8777 as the centre, return up to 30 districts inside a 220-kilometre radius, detailing institutional delivery rate, and exclude boundary geometry.	Get Districts Within Radius
Draw A radius of 75 km around Visakhapatnam and provide data relevant to child health.	Starting from the centre of Ahmedabad, analyse the indicators Skilled Birth Attendants, Institutional Deliveries, and Female Literacy Rate for all districts within 200 kilometres;	Beginning at the centre of Lucknow, assess Exclusive Breastfeeding, ANC Four or More Visits, and Postnatal Care within 48 Hours for districts within 170 kilometres; provide up to 30 districts and remove boundary geometry.	Get Districts Within Radius
For vaccination, return districts up to 110 km from	Taking 17.3850, 78.4867 as the reference point, provide up to 25 districts	Using the centre of Kolkata, compare Full Immunization Coverage, Measles-Rubella	Get Districts Within Radius

Patna.	within 140 kilometres, reporting neonatal mortality rate, and omit boundary shapes.	Vaccination, and DPT3 Coverage for all districts inside 240 kilometres; cap results at 35 districts and omit boundary geometry.	
In relation to noncommunicable diseases, give districts lying within 95 km of Bhopal.	From Ahmedabad, within 180 km, list raw health data but limit to 25 districts.	From the centre of Bengaluru, analyse Under-Five Stunting, Wasting, and Low Birth Weight for districts within a 190-kilometre catchment; return up to 34 districts and exclude boundary geometry.	Get Districts Within Radius
Regarding respiratory infections, providedistricts situated within 85 km of Jaipur.	Using 19.0760,72.8777 as the center, list districts inside 250 km with full indicator data.	Using 11.0168, 76.9558 as centre, return up to 30 districts within 160 kilometres, covering exclusive breastfeeding, and suppress boundary geometry.	Get Districts Within Radius
Districts where diabetes prevalence more than 15.	For 2021, list districts where diabetes prevalence > 15 and vaccination coverage < 70.	For 2021, in Uttar Pradesh, Bihar, and Jharkhand, return up to 120 districts that satisfy neonatal mortality rate ≥ 25, institutional delivery rate ≤ 60,	Get Districts By Constraints

		and ANC four or more visits ≤ 50; omit boundary geometry.	
Point out districts with value in single digit in case of stunting.	In Maharashtra and Gujarat, find districts with stunting ≥ 30 and wasting ≥ 15.	Using 2016 data for Kerala and Tamil Nadu, list up to 200 districts with anemia among women ≥ 55, low birth weight ≥ 20, and exclusive breastfeeding ≤ 40; exclude boundary geometry.	Get Districts By Constraints
Districts where hypertension prevalence more than 25 and BP control among known hypertensives less than 50.	Return up to 150 districts that meet hypertension prevalence ≥ 25 and BP control ≤ 50.	For 2021 across Maharashtra, Gujarat, and Madhya Pradesh, provide up to 180 districts meeting vaccination coverage ≥ 90, measles-rubella vaccination ≥ 85, DPT3 coverage ≥ 85, and diarrhea ≤ 5; no boundary geometry.	Get Districts By Constraints
Districts meeting higher rate on vaccination and lower on diarrhoea.	Exclude boundary geometry and list districts with anemia among women ≥ 55 and modern contraceptive use ≤ 45.	In 2016 for Rajasthan and Madhya Pradesh, list up to 160 districts satisfying diabetes prevalence ≥ 12, hypertension prevalence ≥ 25, and tobacco use among adults ≥ 30; omit	Get Districts By Constraints

		boundary geometry.	
Districts where neonatal mortality rate ≥ 25.	For 2016, find districts where diarrhea prevalence among under-fives ≥ 10 and exclusive breastfeeding ≤ 40.	For 2021 nationwide, return up to 300 districts that meet under-five stunting ≥ 25, wasting ≥ 15, low birth weight ≥ 18, exclusive breastfeeding ≤ 45, and institutional delivery rate ≤ 80; exclude boundary geometry.	Get Districts By Constraints
Show top districts nationally for vaccination coverage.	Provide top 10 districts for full immunization coverage in 2021.	For 2021, across Uttar Pradesh, Bihar, and Jharkhand, report both the top 10 and bottom 10 districts for under-five stunting and wasting.	Get Top Bottom Districts
Identify bottom districts nationally for diabetes prevalence.	List the bottom 5 districts for neonatal mortality rate.	In 2016, identify the bottom 15 districts nationwide for diabetes prevalence and hypertension prevalence and exclude boundary geometry.	Get Top Bottom Districts
List top districts for institutional delivery rate.	Show top districts for institutional delivery rate in Kerala and Tamil	For 2021, provide the top 12 districts for antenatal care four or more visits and postnatal	Get Top Bottom Districts

	Nadu.	care within 48 hours in Maharashtra and Gujarat.	
Indicate bottom districts for under-five stunting.	Return the top 8 districts for modern contraceptive use for 2016.	Return both top 10 and bottom 10 districts for exclusive breastfeeding, low birth weight, and neonatal mortality rate in 2021.	Get Top Bottom Districts
Provide top districts for exclusive breastfeeding.	Present the bottom 12 districts for tuberculosis prevalence and omit boundary geometry.	Show the top 20 districts for vaccination coverage, measles–rubella vaccination, and DPT3 coverage in Andhra Pradesh, Telangana, and Karnataka for 2021, and omit boundary geometry.	Get Top Bottom Districts
Evaluate values in diabetes prevalence between two years	Provide a state-level change analysis for diabetes prevalence in Karnataka.	For Tamil Nadu, deliver a state-level change analysis of exclusive breastfeeding across 2016–2021 and suppress boundary geometry in the outputs.	Get Indicator Change Analysis
Assess how vaccination coverage shifted	Show district-level change in malnutrition for Mumbai between NFHS	Provide a district-level trend assessment of postnatal care within 48 hours for	Get Indicator Change Analysis

nationally.	4 and 5.	Thiruvananthapuram over 2016–2021, including boundary geometry for mapping.	
Summarize the national change in child stunting.	Conduct a state-level assessment of vaccination coverage in Kerala.	Produce a state-level change evaluation for tuberculosis prevalence in Maharashtra spanning 2016–2021 and disable boundary data.	Get Indicator Change Analysis
Review country-level difference in trends in hypertension prevalence.	Report district-level trends in neonatal mortality rate for Jaipur over 2016–2021.	Generate a district-level change profile for low birth weight in Kolkata from 2016 to 2021, retaining geometry for visualization.	Get Indicator Change Analysis
Describe national change in institutional delivery rate.	Present a state-level change summary for anemia among women in Bihar.	Compile a state-level change report for modern contraceptive use in Uttar Pradesh across 2016–2021, and omit boundary geometry while keeping chart outputs.	Get Indicator Change Analysis
Compare Mumbai and Delhi on diabetes	For 2021, compare Mumbai, Delhi, and Kolkata on diabetes	For 2021, compare Mumbai, Delhi, Kolkata, and Bengaluru Urban on diabetes prevalence,	Get District Performance Comparison

prevalence.	prevalence and hypertension prevalence.	hypertension prevalence, and tobacco use among adults against national benchmarks, and omit boundary geometry.	
Benchmark Chennai and Hyderabad for full immunization coverage.	Benchmark Chennai, Hyderabad, and Coimbatore against state averages for institutional delivery rate and ANC four or more visits.	Using state averages in 2021, benchmark Chennai, Hyderabad, Pune, Ahmedabad, and Surat across full immunization coverage, DPT3 coverage, and measles–rubella vaccination, and exclude boundary geometry.	Get District Performance Comparison
Compare Jaipur and Indore on institutional delivery rate.	Using national benchmarks, compare Jaipur, Jodhpur, and Udaipur on stunting and wasting.	For 2016, compare Jaipur, Ajmer, Kota, Udaipur, and Bikaner on institutional delivery rate, ANC four or more visits, and postnatal care within 48 hours against national benchmarks, keeping boundary geometry included.	Get District Performance Comparison
Assess Kolkata and Bengaluru Urban for	For 2016, compare Varanasi, Prayagraj, and Gorakhpur on measles–	In 2021, assess Thiruvananthapuram, Ernakulam, Kozhikode, and	Get District Performance Comparison

tuberculosis prevalence.	rubella vaccination.	Kollam on exclusive breastfeeding, low birth weight, and neonatal mortality rate versus state averages, and omit boundary geometry.)	
Contrast Lucknow and Kanpur Nagar on exclusive breastfeeding.	Against state averages, assess Pune, Nagpur, and Nashik for postnatal care within 48 hours.	For 2021, benchmark Lucknow, Kanpur Nagar, Varanasi, Agra, and Meerut against national benchmarks on modern contraceptive use, institutional delivery rate, and postnatal care within 48 hours, with boundary geometry included.	Get District Performance Comparison
Identify top districts nationally in healthcare performance.	As of 2021, list the top 15 districts in the nutrition category.	For 2021, return both the top 12 and bottom 12 districts in the nutrition category and omit boundary geometry.	Get Multi Indicator Performance
Show bottom districts in the maternal health category.	For 2016, report the bottom 10 districts in maternal health.	Using 2016 data, provide the bottom 15 districts nationwide by a composite of diabetes prevalence, hypertension prevalence, and tobacco use	Get Multi Indicator Performance

		among adults, including boundary geometry.	
List top districts for the nutrition category.	Compare Mumbai, Delhi, and Chennai in the healthcare category composite.	Compare Jaipur, Jodhpur, Kota, and Udaipur on a composite of exclusive breastfeeding, institutional delivery rate, and antenatal care four or more visits for 2021, and omit boundary geometry.	Get Multi Indicator Performance
Indicate bottom districts for the mortality category.	Return the top 8 districts for a composite built from diabetes prevalence and hypertension prevalence.	For 2021, list the top 20 districts by a composite across vaccination coverage, measles–rubella vaccination, and DPT3 coverage, and exclude boundary geometry.	Get Multi Indicator Performance
Provide leading districts in overall healthcare composite.	In 2021, show the top 10 districts in the mortality category.	Using 2021 values, evaluate Bengaluru Urban, Kolkata, Mumbai, and Delhi in the maternal health category composite and include boundary geometry.	Get Multi Indicator Performance
Compare state-	For 2016, evaluate	As of 2021, compare Punjab,	Get State

level performance in healthcare and list each state's leading districts.	maternal health across states and list each state's top districts.	Haryana, Rajasthan in healthcare and return both the top 5 and bottom 5 districts for each state, omitting boundary geometry.	Multi Indicator Performance
Profile nutrition performance across states and highlight leading districts.	Focus on Kerala and Tamil Nadu in the nutrition category and show their leading districts.	Using 2016 data, assess nutrition for Uttar Pradesh, Bihar, Jharkhand and list top 4 districts per state, keeping boundary geometry included.	Get State Multi Indicator Performance
Summarize maternal health performance by state.	Identify the lowest districts in each state for healthcare performance.	For 2021, across Kerala and Karnataka, evaluate exclusive breastfeeding, institutional delivery rate, antenatal care four or more visits, showing both top and bottom 4 districts per state.	Get State Multi Indicator Performance
Assess mortality domain across states, indicating which districts lead within each state.	Return the top 3 districts per state for mortality indicators in 2021.	Compare Assam, Meghalaya, Nagaland on mortality in 2021, surface the bottom 5 districts per state, and omit boundary geometry.	Get State Multi Indicator Performance

Using vaccination coverage, DPT3 coverage, measles–rubella vaccination, compare states and show their best districts.	In Maharashtra and Gujarat, highlight leading districts for diabetes prevalence and hypertension prevalence.	In 2016, for Maharashtra and Telangana, rank top 5 districts per state using a composite of diabetes prevalence, hypertension prevalence, tobacco use among adults, and include boundary geometry.	Get State Multi Indicator Performance
Identify districts with comparable trends in nutrition.	Within Kerala and Tamil Nadu, find districts with similar nutrition patterns.	For 2021, across Karnataka, Maharashtra, and Goa, identify 20 districts with different patterns in nutrition, and exclude boundary geometry.	Get District Similarity Analysis
Find districts that differ in performance across indicators.	Detect districts with different patterns in maternal health in 2021.	Compare Punjab, Haryana, Rajasthan to find 15 districts with similar patterns across exclusive breastfeeding, institutional delivery rate, antenatal care four or more visits, keeping geometry included.	Get District Similarity Analysis
Surface districts	Using 2016 data, identify	Using 2016 data in Assam and	Get District

with similar patterns in maternal health.	districts with similar healthcare patterns for Child Stunting.	West Bengal, detect different patterns for tuberculosis prevalence, tobacco use among adults, returning 10 districts and omitting geometry.	Similarity Analysis
Map districts that are contrasting on mortality indicators.	Select up to 12 districts that are similar on mortality indicators.	Nationwide in 2021, cluster 20 districts that are similar on under-five stunting, wasting, low birth weight, and retain geometry for mapping.	Get District Similarity Analysis
Identify districts with related characterstics on diabetes prevalence, hypertension prevalence, tobacco use.	Find districts that are similar across diabetes prevalence, hypertension prevalence, BP control among known hypertensives, and omit boundary geometry.	For Kerala, Tamil Nadu, and Karnataka, identify different patterns within healthcare, limit to 18 districts, and omit boundary geometry. (category_name + state_names + analysis_type=different + n_districts=18 + include_boundary_data=false)	Get District Similarity Analysis
National classification of	For 2016, classify districts nationally by	For 2016, within Rajasthan, Madhya Pradesh, and	Get District Classification

vaccination coverage performance across districts.	exclusive breastfeeding.	Chhattisgarh, classify districts by neonatal mortality rate and omit boundary geometry.	
Produce a nationwide classification for diabetes prevalence at the district level.	Within Maharashtra and Gujarat, classify districts by anemia among women.	Using 2021 values, classify districts in Kerala and Tamil Nadu by exclusive breastfeeding and include boundary geometry.	Get District Classification
Classify districts nationally by under-five stunting.	Classify districts nationally by tuberculosis prevalence and omit boundary geometry.	For 2016, classify districts across West Bengal, Odisha, and Chhattisgarh by tuberculosis prevalence, omitting boundary geometry.	Get District Classification
Create a national classification for institutional delivery rate.	For 2021, classify districts by modern contraceptive use.	Using 2021 data, classify districts across Andhra Pradesh, Telangana, and Karnataka by full immunization coverage, with boundary geometry included.	Get District Classification
Provide a countrywide	Within Uttar Pradesh, Bihar, and Jharkhand,	For 2016, within Assam, Meghalaya, and Nagaland,	Get District Classification

classification of neonatal mortality rate across districts.	classify districts by low birth weight.	classify districts by hypertension prevalence and omit boundary geometry.	

System-wide Handling of Derived Statistics, Orchestration, and Visualization Logic

The system architecture explicitly separates data storage, statistical computation, LLM-mediated orchestration, and visualization rendering to ensure transparency, reproducibility, and modularity across different query types. Health indicator prevalence values (e.g., diabetes or hypertension) are stored in the database at the district level and retrieved directly through spatial or attribute-based queries. Derived statistics such as state-level averages, inter-district comparisons, percentage differences, and summary metrics are not pre-computed or stored in the database. Instead, these values are calculated dynamically by predefined backend Python functions after data retrieval.

The computation of such derived metrics is fully deterministic and system-defined. While the LLM is responsible for interpreting the user's natural-language query, extracting relevant parameters, and invoking the appropriate backend function, it does not plan or control the statistical computation logic itself. All aggregation, comparison, and normalization operations are implemented as fixed backend routines, ensuring consistent behavior across repeated queries and preventing variability in numerical outputs due to generative processes.

Visualization outputs are produced independently of both the LLM and the backend computation logic. The backend returns structured data (e.g., tabular values, geographic geometries, and computed statistics), which are then consumed by dedicated frontend React components. Each

output modality: textual summaries, charts, and maps, has its own predefined visualization configuration, including selected fields, visualization types (e.g., bar charts, comparative plots, choropleth maps), and color schemes. This design enables consistent visual encoding across queries and indicators.

Overall, this separation of concerns allows the system to support multiple query types and output formats in a scalable manner, while maintaining clear boundaries between LLM-based intent understanding, deterministic data processing, and presentation-layer visualization.

Additional features of the chatbot

Get multi indicator performance

Figure 8 presents the chatbot's output for the prompt: "Benchmark institutional delivery rate, antenatal care four or more visits, and postnatal care within 48 hours for Chennai, Hyderabad, Pune, and Ahmedabad against their respective state averages." As shown in Figure 8(A), it provides the textual breakdown of the list that compares each district's institutional delivery rate, antenatal care four or more visits, and postnatal care to their respective state average. Figure 8(B) represents the bar chart that highlights the performance gaps between each district and its state average across the three maternal health indicators. Complementing these results, Figure 8(C) displays the geographic distribution of the selected districts. The map situates Chennai, Hyderabad, Pune, and Ahmedabad within their respective state contexts, providing spatial orientation for the comparison. The integration of visualized performance gaps with their geographic locations allows users to contextualize how these urban districts outperform their states, offering clearer insights into regional disparities and urban health advantages.

Figure 8. The chatbot's output for the prompt: "Benchmark institutional delivery rate, antenatal

care four or more visits, and postnatal care within 48 hours for Chennai, Hyderabad, Pune, and Ahmedabad against their respective state averages.” (A) Text summary comparing district-level values with state averages, highlighting gaps in institutional delivery, antenatal care, and postnatal care. (B) Bar chart showing performance gaps across districts relative to their state averages for the three maternal health indicators. (C) Interactive map visualizing the geographic distribution of the selected districts and their relative performance compared to state benchmarks.

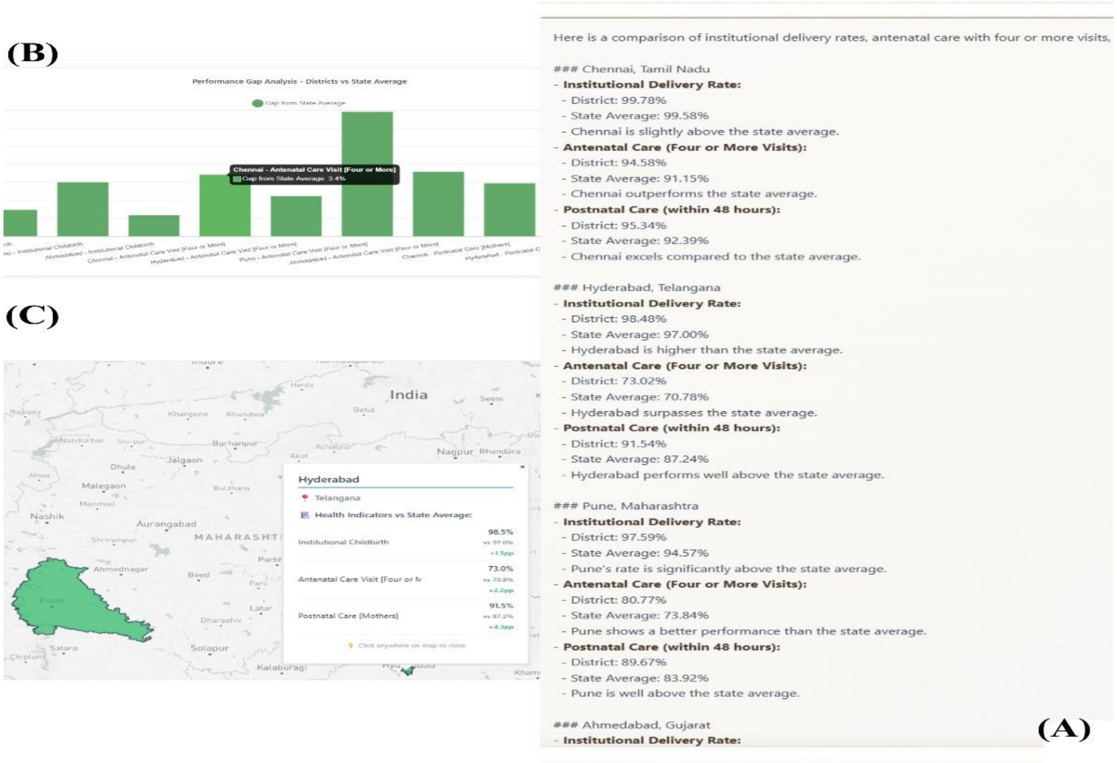

Get border districts

Figure 9 presents the chatbot's output for the prompt: "Examine diabetes prevalence and hypertension prevalence in districts along the Karnataka-Tamil Nadu border, comparing each district with the average of its own state." The chatbot responds to the core question of who leads and who lags within each state. The textual breakdown (Figure 9(A)) organizes detailed values for each district against its state average in a structured format, enabling users to quickly interpret performance metrics. The comparative bar chart (Figure 9(B)) provides a visual summary of prevalence levels across districts, helping users identify differences in performance briefly. Finally, the interactive map (Figure 9(C)) situates the selected border districts geographically, allowing users to contextualize the observed patterns in relation to their location along the Karnataka–Tamil Nadu boundary. The combined presentation of text, charts, and maps ensures that users receive both precise numerical outputs and a broader geographic perspective in a single query.

Figure 9. The chatbot's output for the prompt: "Examine diabetes prevalence and hypertension prevalence in districts along the Karnataka–Tamil Nadu border, comparing each district with the average of its own state." (A) Text summary presenting district-level prevalence of hypertension and diabetes alongside state averages, structured in a bullet-point format for clarity. (B) Bar chart visualizing district performance relative to the state benchmark for both indicators across multiple border districts. (C) Interactive map displaying the spatial distribution of the selected districts along the Karnataka–Tamil Nadu boundary, using color coding to highlight comparative performance.

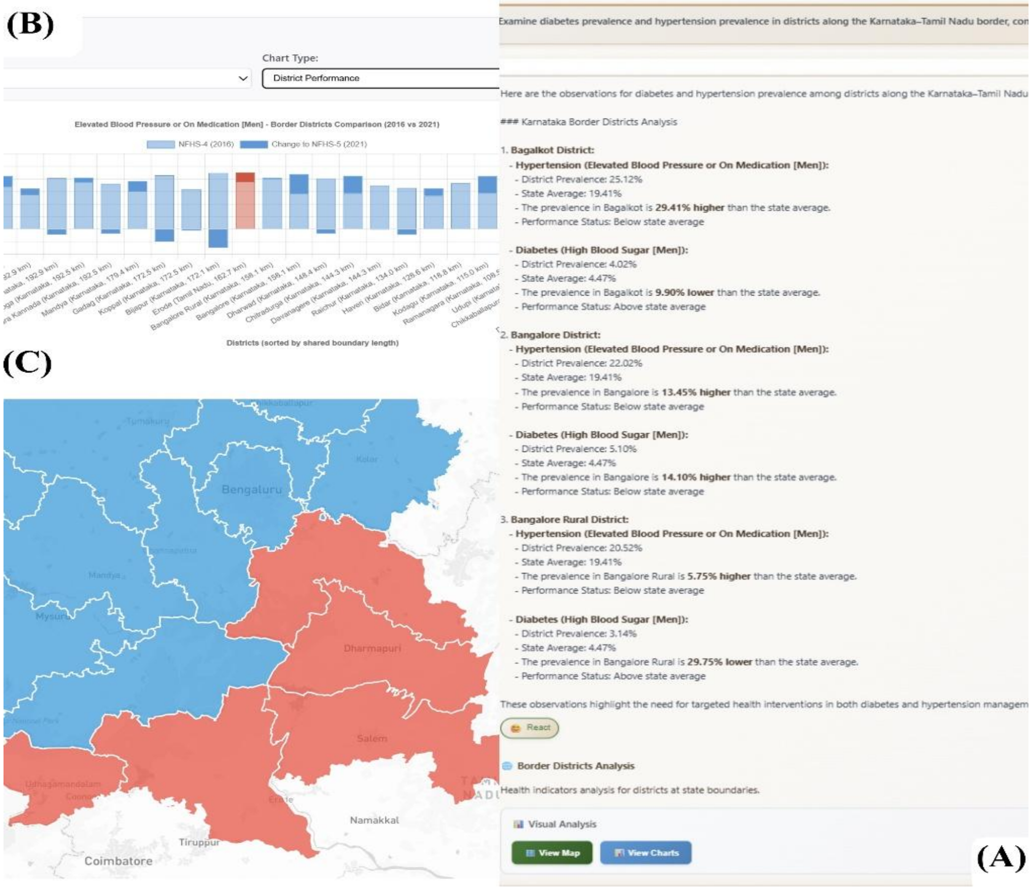

Get districts by constraints

Figure 10 presents the chatbot's output for the prompt: "Can you tell me districts that have population with BPL cards greater than 94?" The chatbot responds to the core question of who leads and who lags within each state. The textual summary (Figure 10(A)) provides district names, corresponding values, and improvements over time in a clear, bullet-point format. The bar chart (Figure 10(B)) offers a visual comparison of BPL card coverage across multiple districts, showing which exceed the defined threshold. Finally, the interactive map (Figure 10(C)) situates the qualifying districts geographically, with markers and pop-up details enhancing spatial context. Together, these outputs combine textual precision, graphical clarity, and geographic visualization to support easy interpretation of constraint-based queries.

Figure 10. The chatbot's output for the prompt: "Can you tell me districts that have population with BPL cards greater than 94?" (A) Text summary listing districts where the share of population with BPL cards exceeds the specified threshold, including percentage values and changes over time. (B) Bar chart displaying district-level BPL card coverage against the threshold criterion. (C) Interactive map highlighting the geographic distribution of the qualifying districts, with pop-up details for selected locations.

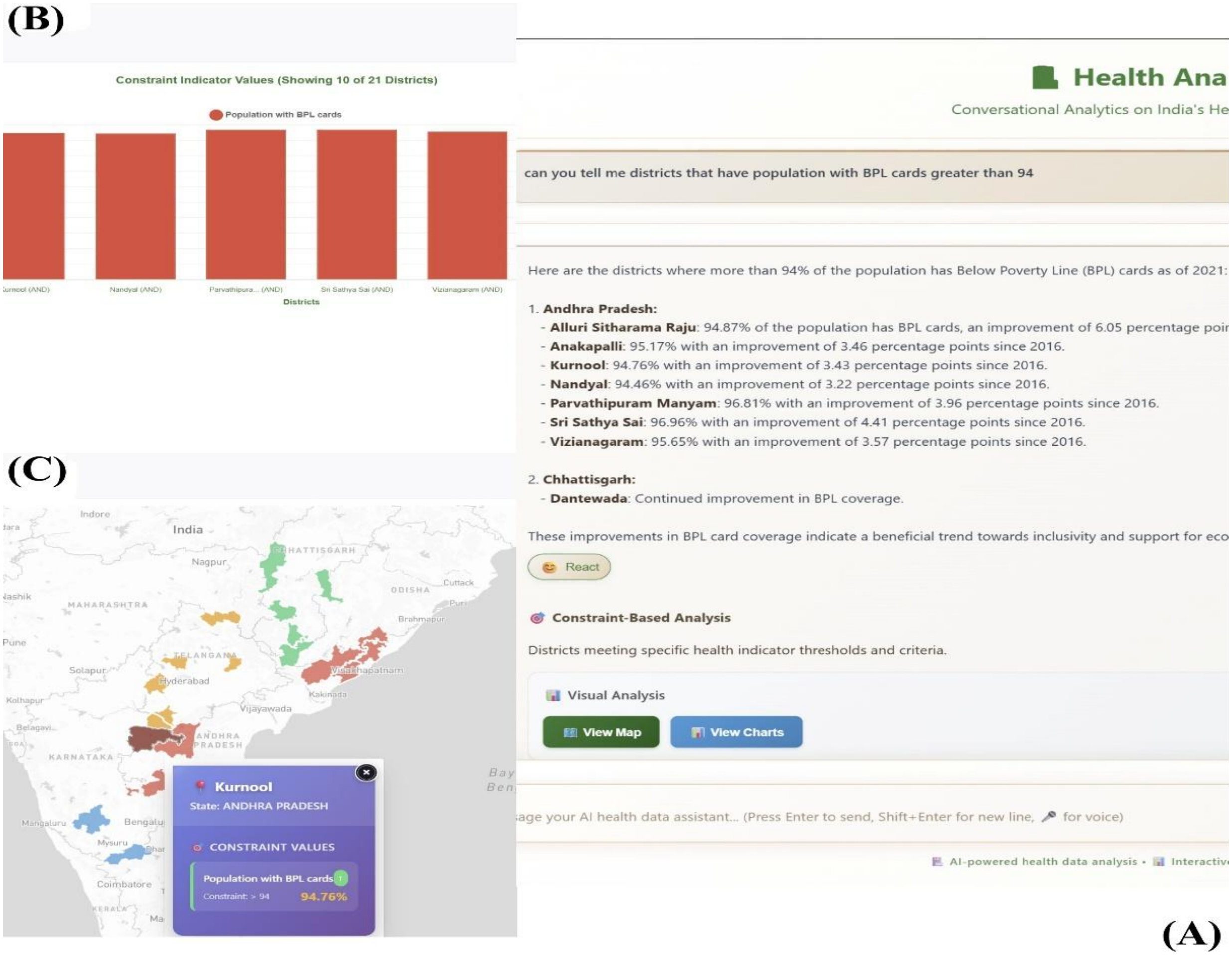

Get state-wise indicator extremes

As shown in Figure 11(A), the textual summary lists, for each state, the best and worst performing districts for the selected indicator, along with their percentage values, trends over time, and whether performance is improving or declining. Complementing the text, bar charts offer comparative perspectives. One chart contrasts the best and worst performing districts across all states, while another highlights intra-state disparities by displaying the performance gap between them (Figure 11(B)). These visualizations facilitate quick identification of extremes and assessment of relative variation. In addition, a geographic map (Figure 11(C)) plots the locations of the best and worst performers, using green to represent the best and red to represent the worst. Interactive pop-ups provide detailed attribute information, including district name, current value, trend, and performance direction. Taken together, these outputs deliver both detailed numerical data, descriptive summary and broader spatial insights, enabling users to clearly understand where performance is strongest and weakest across the country.

Figure 11. The chatbot’s output for the prompt: “Now provide me the best and worst performing district in all states based on Population with BPL cards.” (A) Text summary listing the best- and worst-performing districts in each state, including percentage values, trends, and performance directions. (B) Bar charts showing intra-state comparisons and cross-state contrasts of best versus worst districts. (C) Geographic map highlighting the best- and worst-performing districts across states.

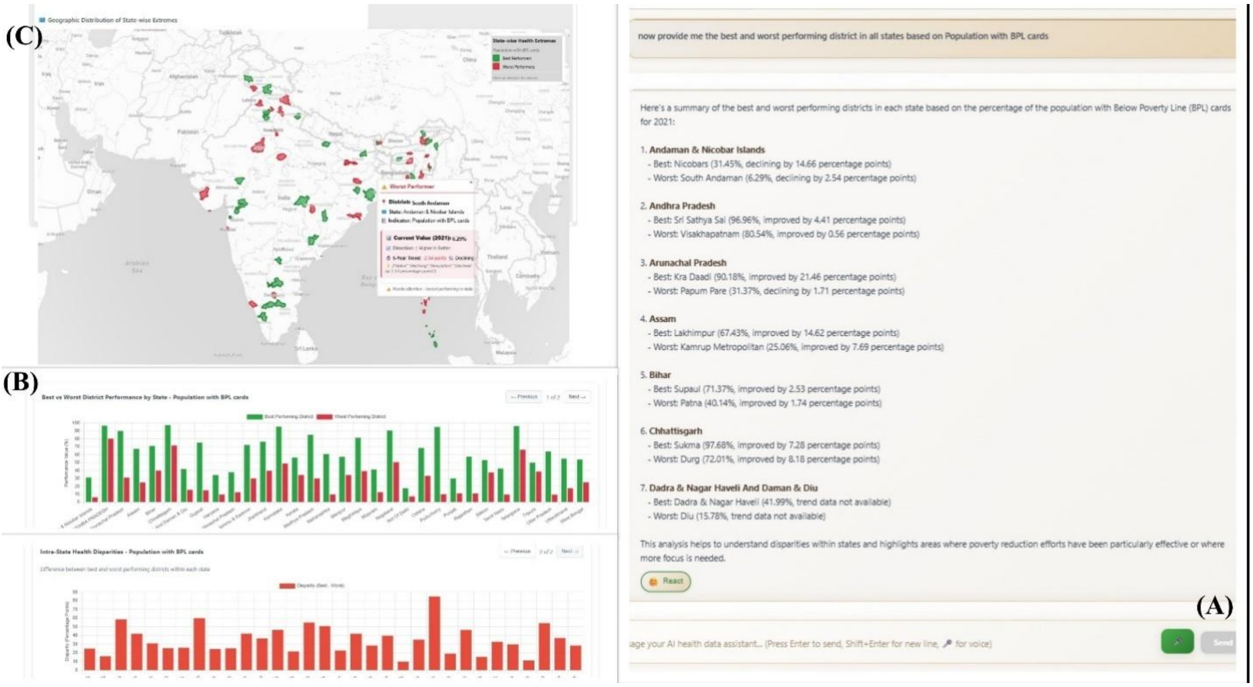

Get District Classification

The chatbot (Figure 12(A)) provides a clear categorization of each district into groups such as Very Low, Low, Moderate, or High, along with the corresponding indicator values. To complement this, a bar chart (Figure 12(B)) summarizes the number of districts within each category, making it easy to compare performance levels across the region. Finally, the classification map (Figure 12(C)) applies a color-coded scheme to illustrate how districts fall into different categories, helping to identify regional clusters of high or low performance at a glance. Together, these outputs enable users to interpret both detailed district-level statistics and broader spatial patterns of performance.

Figure 12. The chatbot's output for the prompt: "Classify the districts in West Bengal based on their child stunting values." (A) Text summary categorizing districts into Very Low, Low, Moderate, or High stunting groups with corresponding indicator values. (B) Bar chart showing the number of districts within each performance group. (C) Color-coded classification map illustrating regional clustering of district performance.

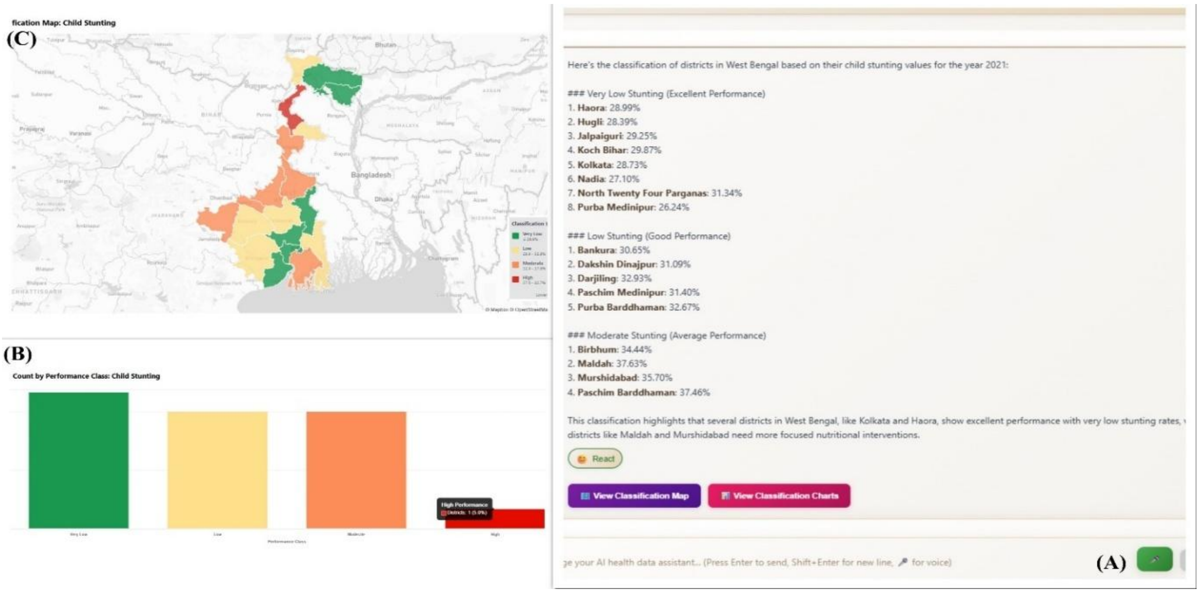

References

Downie, Nick, and Chart.js Team. 2025. “Chart.Js.” npm. Accessed August 27. https://www.chartjs.org/docs/latest/.

Ramírez, Sebastián. 2025. “FastAPI.” Python web framework. Accessed August 5. https://fastapi.tiangolo.com/.

vis.gl. 2025. “Home | React-Map-Gl.” npm. Accessed August 27. https://visgl.github.io/react-map-gl/.

Hertzen, Niklas von. 2025. “Html2canvas - Npm.” npm. Accessed August 27. https://www.npmjs.com/package/html2canvas.

Mapbox. 2025. “Mapbox GL JS | Mapbox.” npm. Accessed August 27. https://docs.mapbox.com/mapbox-gl-js/guides/.

OpenAI. 2024. “Function Calling - OpenAI API.” Accessed November 29. https://platform.openai.com/docs/guides/function-calling.

OpenAI, and Shantanu Jain. 2025. “Tiktoken.” OpenAI. Accessed August 24. https://github.com/openai/tiktoken.

PostGIS Development Group. 2025. “PostGIS.” Accessed August 5. https://postgis.net/.

Gregorio, Federico Di. 2025. “Psycopg.” PostgreSQL database adapter for Python. Accessed August 31. https://www.psycopg.org/docs/.

Ayerst, Jeremy, and react-chartjs-2 Team. 2025. “React-Chartjs-2.” npm. https://react-chartjs-2.js.org/.

Meta Platforms Inc. 2025. “React.” Accessed August 5. https://opensource.fb.com/projects/react/.

Redocly. 2025. “ReDoc.” Open-source API documentation generator. Accessed August 30. https://github.com/Redocly/redoc.

Su, Qi, and Jennifer Widom. 2005. "Indexing Relational Database Content Offline for Efficient Keyword-Based Search." *Proceedings of the International Database Engineering and Applications Symposium, IDEAS* 2005-Janua (January). Institute of Electrical and Electronics Engineers Inc.: 297–306. doi:https://doi.org/10.1109/IDEAS.2005.36.

SmartBear Software. 2025. "Swagger Editor." Accessed August 30. https://editor.swagger.io/.

The PostgreSQL Global Development Group. 2025a. "PostgreSQL: Documentation: 17: 64.2. GiST Indexes." Accessed August 31. https://www.postgresql.org/docs/current/gist.html.

———. 2025b. "PostgreSQL: The World's Most Advanced Open Source Database." Accessed August 5. https://www.postgresql.org/.

Colvin, Samuel. 2025. "Pydantic." Python data-validation-library. Accessed August 30. https://docs.pydantic.dev/latest/.
